# Mediator Directs Co-transcriptional Heterochromatin Assembly by RNA Interference-Dependent and -Independent Pathways

**DOI:** 10.1371/journal.pgen.1003677

**Published:** 2013-08-15

**Authors:** Eriko Oya, Hiroaki Kato, Yuji Chikashige, Chihiro Tsutsumi, Yasushi Hiraoka, Yota Murakami

**Affiliations:** 1 Laboratory of Bioorganic Chemistry, Department of Chemistry, Faculty of Science, Hokkaido University, Sapporo, Japan; 2 Department of Biochemistry, Shimane University School of Medicine, Izumo, Japan; 3 PRESTO, Japan Science and Technology Agency (JST), Honcho Kawaguchi, Saitama, Japan; 4 National Institute of Information and Communications Technology, Kobe, Japan; 5 Graduate School of Frontier Biosciences, Osaka University, Suita, Japan; Duke University, United States of America

## Abstract

Heterochromatin at the pericentromeric repeats in fission yeast is assembled and spread by an RNAi-dependent mechanism, which is coupled with the transcription of non-coding RNA from the repeats by RNA polymerase II. In addition, Rrp6, a component of the nuclear exosome, also contributes to heterochromatin assembly and is coupled with non-coding RNA transcription. The multi-subunit complex Mediator, which directs initiation of RNA polymerase II-dependent transcription, has recently been suggested to function after initiation in processes such as elongation of transcription and splicing. However, the role of Mediator in the regulation of chromatin structure is not well understood. We investigated the role of Mediator in pericentromeric heterochromatin formation and found that deletion of specific subunits of the head domain of Mediator compromised heterochromatin structure. The Mediator head domain was required for Rrp6-dependent heterochromatin nucleation at the pericentromere and for RNAi-dependent spreading of heterochromatin into the neighboring region. In the latter process, Mediator appeared to contribute to efficient processing of siRNA from transcribed non-coding RNA, which was required for efficient spreading of heterochromatin. Furthermore, the head domain directed efficient transcription in heterochromatin. These results reveal a pivotal role for Mediator in multiple steps of transcription-coupled formation of pericentromeric heterochromatin. This observation further extends the role of Mediator to co-transcriptional chromatin regulation.

## Author Summary

DNA is packaged into chromatin structure, which is important for various genome functions such as gene expression and maintenance of genetic information. Heterochromatin is a condensed chromatin structure and involved in epigenetic regulation of gene expression through repression of transcription. Heterochromatin at the pericentromeric repeats in fission yeast is assembled by two distinct mechanisms, RNAi-dependent and Rrp6, a component of the nuclear exosome, -dependent mechanisms. In addition, heterochromatin spreads into neighboring regions in an RNAi-dependent manner. Both mechanisms are coupled with the transcription from the target loci by RNA polymerase II, but the molecular nature of the coupling is not understood. Here we showed that the multi-subunit complex Mediator, which directs initiation of RNA polymerase II-dependent transcription, functions in the coupling between transcription and heterochromatin assembly. Mediator is required for Rrp6-dependent heterochromatin assembly and contributes to the RNAi-dependent spreading of heterochromatin via enhancement of production of siRNA by RNAi machinery. These observations highlight the multi-functions of Mediator in the transcription-coupled processes.

## Introduction

Heterochromatin is a silent higher-order chromatin structure that is associated with various genome functions such as transcriptional regulation, chromosomal segregation, suppression of recombination and repression of selfish elements. The fission yeast *Schizosaccharomyces pombe* provides a good model system for investigating heterochromatin formation. In fission yeast, heterochromatin is preferentially enriched across large chromosomal domains at the pericentromeres, subtelomeres and the mating-type locus. These regions are rich in methylation of histone H3K9 (H3K9me), which is catalyzed by the histone methyltransferase Clr4, a homolog of mammalian SUV39h [1], [2]. The modification of H3K9me is critical for the binding of HP1 proteins [2], [3], which recruit various factors for the assembly of repressive chromatin and associated various functions [4], [5].

Several distinct pathways promote heterochromatin assembly in fission yeast. At the pericentromere, RNAi machinery plays essential roles in heterochromatin formation [6], [7]. Pericentromeric heterochromatin is assembled on the outer repeat (*otr*) region (containing of *dg* and *dh* repeats), and the outer portion of the innermost repeats (*imr*), which surround the central core (*cnt*) domain, the site of kinetochore assembly [8]. The repeats are transcribed by RNA polymerase II (RNAPII) to produce non-coding RNAs (ncRNAs) during S-phase [9], [10], [11]. Transcribed ncRNAs give rise to double-strand RNA via the RNA-dependent RNA polymerase complex (RDRC), comprised of Rdp1, Cid12 and Hrr1, and are processed into small interfering RNAs (siRNAs) by the RNase III helicase Dicer (Dcr1). The siRNAs are then loaded into an RNA-induced transcriptional silencing (RITS) complex composed of Ago1, Tas3 and Chp1 [7]. siRNAs target the RITS complex to cognate nascent transcripts, resulting in the recruitment of additional factors, including RDRC and ultimately Clr4, to methylate histone H3K9. Generation of siRNAs and heterochromatin assembly are interdependent processes that form a self-enforcing loop [12], [13]. Importantly, RNAPII appears to couple transcription at the target loci with the generation of siRNAs. This was shown by the fact that a specific mutation in RNAPII results in a decrease in heterochromatic histone modifications, accumulation of pericentromeric transcripts, and accompanying loss of siRNAs, which are effects that were observed previously in RNAi mutants [10].

Heterochromatin, once established, spreads into neighboring region, which is typically shown by the heterochromatin formation and silencing of the genes inserted into heterochromatin. This process depends on RNAi system and probably couples with transcription [14], [15].

Nuclear RNA is monitored by a nuclear RNA surveillance system involving exosomes with 3′-5′ exonuclease activity, and a portion of the ncRNA at the pericentromere has been shown to be degraded by the nuclear exosome [16], [17]. In addition to RNA degradation, 3′-5′ exonuclease Rrp6, a component of the nuclear exosome, was shown to mediate heterochromatin formation in parallel with RNAi, which is demonstrated by the cumulative increase and decrease of H3K9me at the pericentromere in the double null-mutant of *ago1* and *rrp6* [18]. Since the amount of siRNA is not affected by depletion of Rrp6, Rrp6-dependent heterochromatin formation occurs via a pathway that is distinct from that of RNAi-dependent siRNA generation [16]. The molecular basis of the Rrp6-dependent pathway is not yet clear.

The *cenH* sequence, which shows 96% homology to centromeric *dg* and *dh* repeats, is present at the silent mating-type (*mat2/3*) locus and serves as an RNAi-dependent heterochromatin nucleation center [6], [19]. In parallel with the RNAi-dependent pathway, the ATF/CREB family DNA-binding proteins, Atf1 and Pcr1, participate in heterochromatin nucleation with a histone deacetylase, Clr3 [20].

Mediator, which is a well-conserved protein complex consisting of at least 20 subunits, was first identified as a factor that mediates DNA transcription factors binding at regulatory sequneces and RNAPII at promoters for the efficient start of transcription [21], [22] and has been shown to be required for transcription of almost all protein-coding genes *in vivo* [23], [24], [25]. Structural analysis indicates that this complex consists of four distinct structural domains: head, middle, tail and kinase. The head domain is responsible for extensive interaction with RNAPII, and the Med18/Pmc6-Med20 heterodimer, which is a portion of the head domain, binds to the core head domain through the C-terminal helix of Med8 [26]. The head domain stabilizes the connection between RNAPII and TFIIH, which facilitates the transition from initiation complex to elongation complex [27]. In addition to the promotion of general transcription from protein-coding genes, recent studies have revealed a new function of Mediator. In *Arabidopsis thaliana*, Mediator directs the transcription of ncRNA genes by recruiting RNAPII to their promoters [28]. In mammalian cells, a specific subunit of Mediator functions as an interaction site for alternative mRNA splicing or transcription elongation factors [29], [30]. These data suggest that Mediator might play roles in both transcription elongation and the subsequent processing of transcripts as a platform for the recruitment of various factors.

Since both Rrp6-dependent heterochromatin formation and RNAi-dependent heterochromatin formation are coupled with transcription, we assumed that the factor(s) that interacts with RNAPII directs the coupling. Therefore, we assessed the role of several RNAPII-interacting factors in pericentromeric heterochromatin assembly. We found that the disruption of Med18 and Med20, non-essential subunits of the Mediator head domain (MHD), compromised both RNAi-dependent and Rrp6-dependent heterochromatin assembly at the pericentromere. In addition, the head domain is required for transcriptional activation in heterochromatin. Therefore, we propose that Mediator links transcription of ncRNA and its processing by RNAi and exosomes for the formation of centromeric heterochromatin.

## Results

### Mediator is required for heterochromatic silencing at the pericentromeres

**Figure 1:**
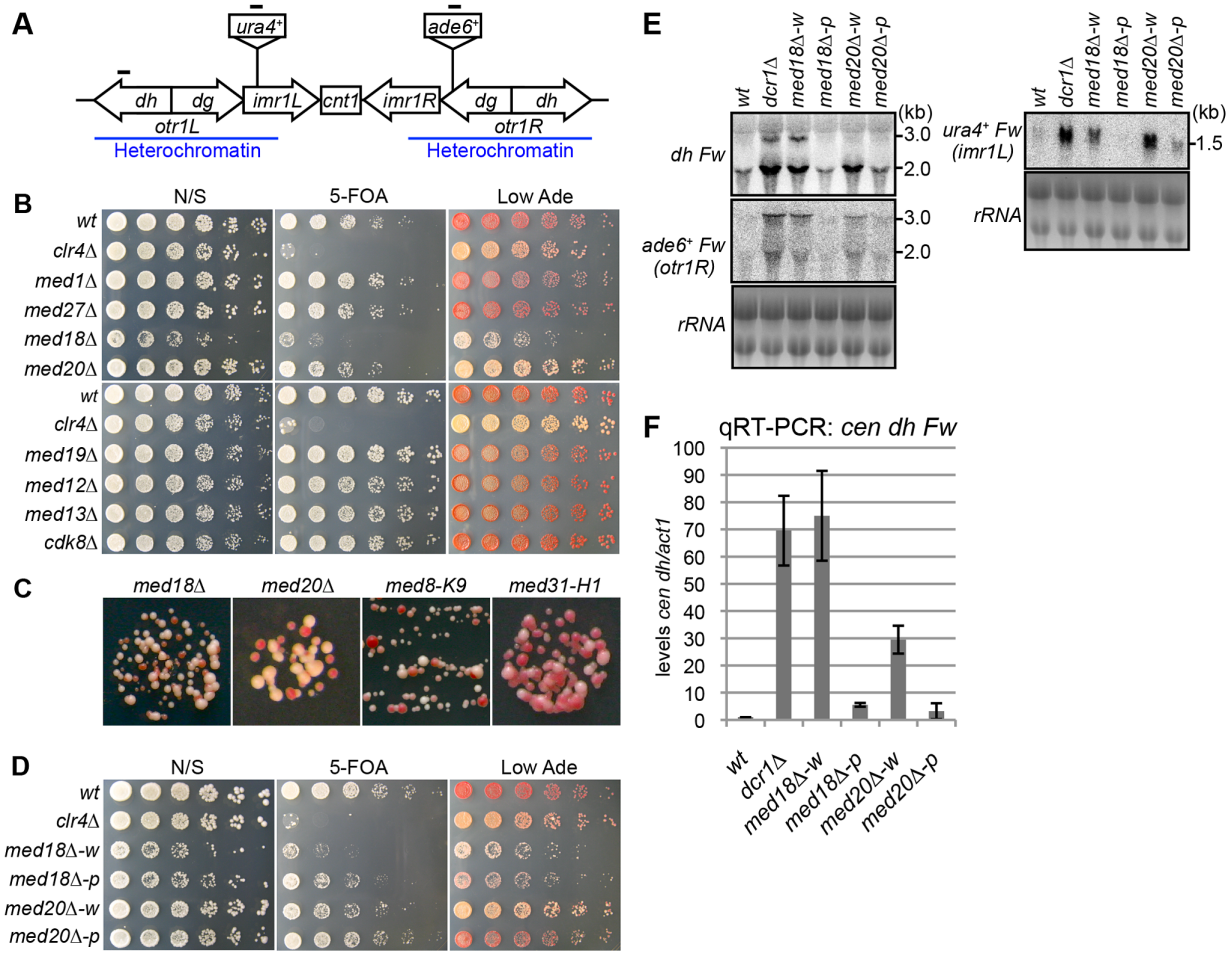
Mediator is required for heterochromatic silencing at the pericentromere. (A) Schematic of fission yeast centromere 1. Locations of *ura4* and *ade6* reporter genes inserted within the pericentromeric region are shown (*imr1L::ura4^+^* and *otr1R::ade6*). Black bars indicate the location of primers or probes used for ChIP, RT-PCR and northern analysis. (B) Silencing assay at the pericentromere. Shown are the results of serial dilutions of the indicated strains spotted onto non-selective media (N/S), medium with 5-fluoroorotic acid (5-FOA), and medium with a limited amount of adenine (Low Ade) to assay *ura4^+^* and *ade6^+^* expression. (C) Spots on Low Ade medium using Mediator mutants (*med18Δ*, *med20Δ*, *med8-K9* and *med31-H1*), which are defective in heterochromatic silencing at the pericentromere. (D) Silencing assay at the pericentromere. Shown are the results of serial dilutions of the indicated strains spotted onto N/S, 5-FOA and Low Ade media to assay *ura4^+^* and *ade6^+^* expression. *w* indicates white epiclones (*med18Δ-w* and *med20Δ-w*), and *p* indicates pink epiclones (*med18Δ-p* and *med20Δ-p*). (E) Northern Analysis of *dh*, *otr1R::ade6* and *imr1L::ura4* forward strand transcripts in wild-type (*wt*) and mutant cells using oligonucleotide probes. rRNA was used as a loading control. (F) Quantitative RT-PCR analysis of *cen dh* forward transcript levels relative to a control *act1^+^*, normalized to the wild type in the indicated strains. Error bars show the standard error of the mean (n = 3).

To investigate whether Mediator is involved in heterochromatin assembly, each gene encoding a non-essential subunit of mediator was disrupted in a strain possessing marker genes in the pericentromeric heterochromatin (*otr1R::ade6^+^* and *imr1L::ura4^+^*) to monitor heterochromatic silencing (Figure 1A) [31]. Since the *otr1R::ade6^+^* and *imr1L::ura4^+^* genes are repressed by heterochromatin, the wild-type strain formed red colonies on a solid medium containing a limiting amount of adenine (Low Ade) and was resistant to 5-fluoroorotic acid (5-FOA), a counter-selective drug for *ura4* ^+^ expression. By contrast, heterochromatin mutants, such as *clr4Δ*, formed white or pink colonies on the Low Ade plate and showed sensitivity to 5-FOA (Figure 1B). Among the eight non-essential subunits of mediator tested (*med1/pmc2*, *med27/pmc3*, *med18/pmc6*, *med20*, *med19*/*rox3*, *med12/srb8*, *med13/srb9* and *cdk8/srb10*), only disruption of *med18* (also known as *pmc6*) and *med20* resulted in the formation of pink colonies and increased sensitivity to 5-FOA (Figure 1B), which is in accordance with growth on plates lacking uracil or adenine (Figure S1A). Closer examination revealed that both *med18Δ med20Δ* cells formed a mixture of white and pink colonies on Low Ade plates. In addition, point mutants of *med8* (*med8-K9*) and *med31* (*med31-H1*), which were isolated by the screening of heterochromatic mutants (Figure S1B; Kato et al. submitted), also formed a mixture of pink and white colonies (Figure 1C, Figure S1C).

The variegation in the color of colonies by the mutation of Mediator subunits suggested that silencing of the *otr1R::ade6^+^* gene was variegated in the mutant cells and that distinct levels of *otr1R::ade6^+^* silencing were epigenetically inherited. To test the stability of the pink and white phenotype, pink and white colonies of each mutant were selected, cultured in YES media overnight and re-spotted onto Low Ade and 5-FOA plates. Re-spotting of the cells from white colonies (*med18Δ-w, med20Δ-w* and *med8-K9-w*) and pink colonies (*med18Δ-p med20Δ-p* and *med8-K9-p*) produced predominantly white colonies and pink colonies, respectively (Figure 1D, Figure S1C). This indicated that the white and pink phenotypes were epigenetically inherited through generation but exchangeable, which is further confirmed by the measurements of the conversion rates between white and pink epiclones (Figure S2). The conversion rates are different in each mutants, but in all mutants, conversion rates from pink to white is higher than those of white to pink, showing that white-epiclones, in which heterochromatin is compromised, are more stable. Hereafter, we designate the epigenetic clones derived from white colonies and red colonies as white and pink “epiclones”, respectively. *med18Δ-w* and *med20Δ-w* showed greater sensitivity to 5-FOA than *med18Δ-p* and *med20Δ-p* (Figure 1D), indicating that silencing at *imr1L::ura4^+^* was also compromised more severely in the white epiclones and that the silencing defect at *otr1R::ade6^+^* is connected with that at *imr1L::ura4^+^*. This suggested that the white phenotype reflected silencing defects of the entire pericentromeric heterochromatin. It should be noted that it was difficult to separate the white epiclones from the pink epiclones of *med31-H1* cells (*med31-H1-w* and *med31-H1-p* in Figure S1C) because of frequent variegation between the two (Figure S2).

The loss of heterochromatic gene silencing was confirmed in Mediator mutants by measuring the accumulation of transcripts from the pericentromeric repeats (*dg* and *dh*) and inserted marker genes. Strand-specific northern analysis showed a large increase in those transcripts in both *med18Δ-* and *med20Δ-w* cells (Figure 1E and Figure S3), which was consistent with the observed silencing defects (Figure 1D), while only marginal accumulation was observed in *med18Δ-p* and *med20Δ-p* cells. Both point mutants of the other Mediator subunits (*med8-K9* and *med31-H1*) also showed accumulation of the transcripts (Figure S3). Accumulation of heterochromatic transcripts from *dh* repeats was also demonstrated by strand-specific RT-PCR (Figure 1F). These results showed that the Mediator subunits Med8, Med18, Med20 and Med31 were involved in silencing of pericentromeric heterochromatin. Med18 and Med20 form a heterodimer that associates with head domain core complex through the C-terminal linker region of Med8 [26], and the connection between the Med18/Med20 heterodimer and head domain appears to be lost in the *med8-K9* mutant (Figure S1B). In addition, Med31, a component of the middle domain, is located close to the head domain. Because these findings indicate that Mediator functions in pericentromeric heterochromatin via the head domain, Med18 and Med20 were selected for closer examination.

### Mediator localizes with RNAPII at the pericentromeric repeats

Both RNAi- and Rrp6-dependent heterochromatin formation, which occur in parallel at the pericentromere, appear to be coupled with the transcription of ncRNA at the pericentromeric repeats [10], [18]. We, hence, assume that Mediator contributed to pericentromeric heterochromatin formation directly through the transcription of ncRNA and/or processing of ncRNA. If this assumption was true, Mediator should localize to the transcribed region in heterochromatic repeats. To test this possibility, the localization of Med20-5Flag and RNAPII to the transcribed regions of *dh* repeats was examined by Chromatin immunoprecipitation (ChIP) assay. Since heterochromatic ncRNA is mainly transcribed during G1/S-phase [11], cell cycle was synchronized using the *cdc25* temperature-sensitive mutation. The results show that RNAPII accumulated during G1 to early S-phase, followed by the accumulation of transcripts (, C, D). Med20-5Flag showed a similar oscillating pattern, but the peak disappeared slightly earlier than the Pol2 peak (Figure S4B). This is consistent with the speculation that Mediator is involved in the heterochromatic ncRNA transcription.

### Loss of Med18 or Med20 causes defects in heterochromatin structure at the pericentromeres

**Figure 2:**
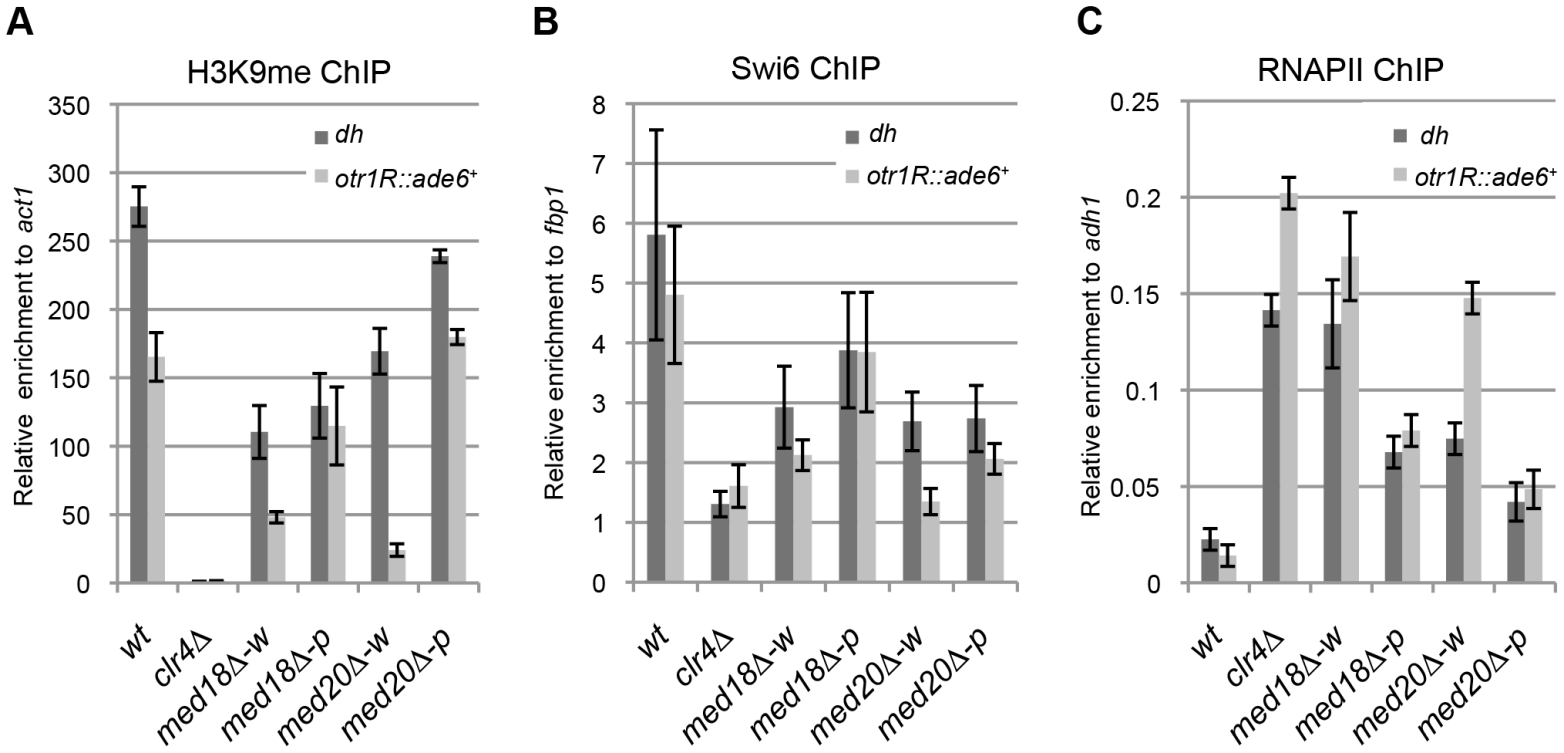
Loss of Med18/20 causes defects in heterochromatin structure at the pericentromere. ChIP analysis of H3K9me (A), Swi6 (B) and RNAPII (C) at *dh* repeats or *otr1R::ade6^+^* relative to *act1*, *fbp1* or *adh1*, respectively. Error bars show the standard error of the mean (n = 3).

To gain further insight into the roles of Mediator during heterochromatin organization, the occupancy of H3K9me2, Swi6 and RNAPII at the centromeric heterochromatin was assessed by ChIP assay. At the inserted marker gene (*otr1R::ade6^+^*), the levels of histone H3K9me and Swi6 were decreased and RNAPII occupancy was increased in the white epiclones of *med18Δ* and *med20Δ* (Figure 2A–C). This indicated that heterochromatin structure at the marker gene was disrupted in the white epiclones. By contrast, in the pink epiclones of *med18Δ* and *med20Δ*, the decrease in H3K9me/Swi6 and increase in RNAPII were less prominent than those in white epiclones. This reflected the difference in silencing defects in each epiclone (Figure 1D). At the heterochromatic repeats, *dh*, H3K9me/Swi6 and RNAPII were also decreased and increased in the Mediator mutants, respectively, but the differences between the white and pink epiclones were less prominent than at *otr1R::ade6^+^*. These results showed that the accumulation of transcripts from heterochromatic repeats and marker genes is, at least in part, due to an increase in transcription induced by the disruption of heterochromatin structure. These results also confirm that Mediator is required for heterochromatin formation at the pericentromere.

### Med18 is required for Rrp6-dependent H3K9 methylation at the pericentromere

**Figure 3:**
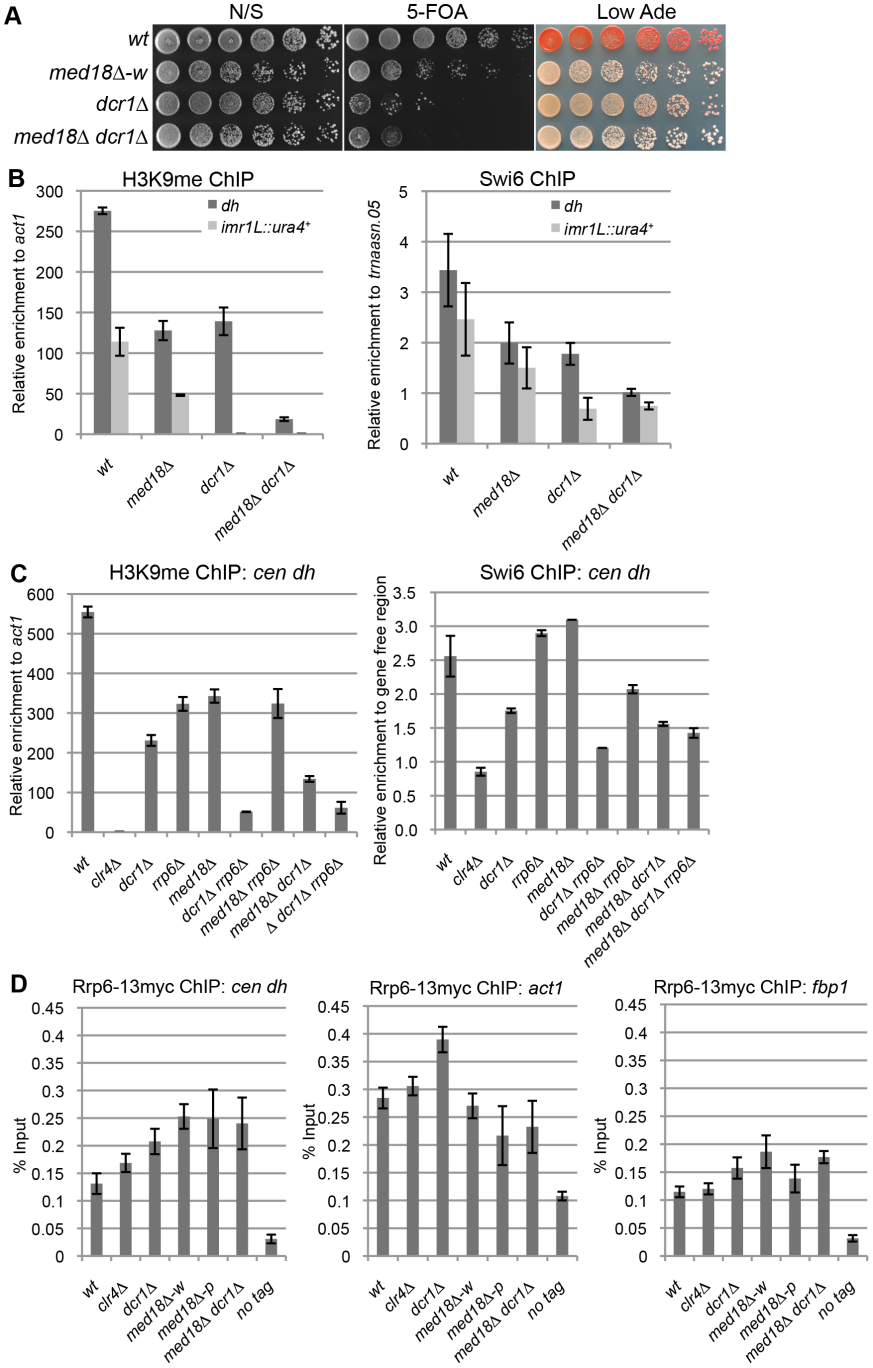
Med18/Mediator is required for Rrp6/Exosome-dependent H3K9 methylation at the pericentromere. (A) Silencing assay at the pericentromere. Shown are the results of serial dilutions of the indicated strains spotted onto N/S, 5-FOA and Low Ade media to assay *ura4^+^* and *ade6* expression. (B) ChIP analysis of H3K9me (left panel) and Swi6 (right panel) at *dh* repeats or *imr1L::ura4^+^* relative to *act1* or a tRNA genes (*trnaasn.05*), respectively. Error bars show the standard error of the mean (n = 3). (C) ChIP analysis of H3K9me (left panel) and Swi6 (right panel) at the *dh* repeats relative to *act1* or a gene-free region, respectively. Error bars show the standard error of the mean (n = 3). (D) ChIP analysis of Rrp6-13myc at *dh* repeats (left panel), *act1* (middle panel) and *fbp1* (right panel). Enrichment relative to the input whole cell extract (WCE) in the indicated strains are shown. Error bars represent the standard error of the mean (n = 3).

There are two distinct pathways for heterochromatin formation at the pericentromeric repeats: RNAi-dependent and Rrp6-dependent pathways. In RNAi mutants such as *dcr1Δ*, H3K9me is diminished at the inserted marker genes but substantially retained at the pericentromeric repeats, while disruption of *rrp6* did not affect H3K9 me at the marker genes [18]. The distribution of H3K9me in the white epiclones of the Mediator mutants resembled that observed in *dcr1Δ* cells; the level of H3K9me at the marker genes was lower than that at heterochromatic repeats. Thus, We speculated that Mediator is involved in the RNAi-dependent pathway. To confirm this, a *med18Δ dcr1Δ* double mutant was established and used to examine heterochromatin silencing and the amount of H3K9me and Swi6 at *dh* repeats and *imr1:: ura4^+^* (Figure 3A, B). Note that since *med18Δ Δdcr1Δ* cells did not exhibit the variegated phenotype observed in the *med18Δ* single mutant (Figure 3A), white and pink epiclones of *med18Δ* cells were not separated in the following experiments (Figure 1B, C and D). If Mediator functions in the RNAi-dependent pathway, the *med18Δ dcr1Δ* double mutants would retain H3K9me and Swi6 to the level similar to those in single mutant. However, in the double mutant, the retained H3K9me/Swi6 at the *dh* repeats was significantly decreased compared to each single mutant (Figure 3B), suggesting that, contrary to our speculation, Med18 functions in a pathway distinct from the RNAi-dependent pathway.

Because the results in Figure 3B were reminiscent of the results reported by Reyes-Trucu et al., in which the amount of H3K9me retained at the centromeric repeats in *ago1Δ* cells was significantly decreased by further disruption of *rrp6* [18], we speculated that Mediator functioned in an Rrp6-dependent heterochromatin formation pathway. To confirm this, single, double and triple mutant cells of *dcr1*, *med18* and *rrp6* were used to measure the amount of H3K9me at the *dh* repeats (Figure 3C, left panel). Each single mutant, as well as the *med18Δ rrp6Δ* double mutants, retained similar amounts of H3K9me. By contrast, combination of *dcr1Δ* with *rrp6Δ* caused a substantial decrease in H3K9me, which was consistent with the previous proposal that both RNAi- and Rrp6-dependent pathways contribute to heterochromatin formation at the pericentromeres [18]. Similarly, the combination of *dcr1Δ* with *med18Δ* also caused a significant decrease in H3K9me, while *med18Δ rrp6Δ* cells maintained a level of H3K9me comparable to each single disruptant. The H3K9me retained in *med18Δ rrp6Δ* cells was also decreased by the introduction of *dcr1Δ*. The amount of Swi6 in each mutant reflects the amount of H3K9me (Figure 3C, left panel). These results clearly indicate that Med18 functions in the same heterochromatin formation pathway as Rrp6 at the *dh* repeats. Therefore, H3K9me in *dcr1Δ* cells was retained by the Rrp6/Med18-dependent pathway, while H3K9me in each of the *med18Δ* and *rrp6Δ* cells was maintained by the RNAi-dependent pathway.

Details of the Rrp6-dependent pathway are not clear yet; even the localization of Rrp6 at heterochromatin has not been examined. We, thereby, analyzed the localization of Rrp6 tagged with myc epitope at heterochromatin (*dh*) as well as euchromatin (*act*1 and *fbp1*) (Figure. 3D). Compered with no-tag control, Rrp6-myc was enriched at both *dh* and euchromatic genes to the same extent. Depletion of *clr4* did not affect the localization of Rrp6-myc, while deletion of *dcr1* caused a slight increase at all loci. The enrichment of Rrp6 at *dh* increased in *med18Δ-w* and *med18Δ-p* epiclones, and also in the *med18Δ dcr1Δ* double mutant, while the enrichment at euchromatic genes was marginally affected in those mutant cells. This suggests that Mediator functions in a step after association of Rrp6 on chromatin for heterochromatin formation.

### Mediator is required for the generation of siRNA from pericentromeric ncRNA

Both *dcr1Δ* and *rrp6Δ* cells retained similar levels of H3K9me at centromeric repeats (Figure 3C). By contrast, deletion of *rrp6* does not affect H3K9me at the marker genes inserted in centromeric repeats [18], whereas deletion of *dcr1* caused the loss of H3K9me on the marker genes (Figure 3B), indicating that the spreading of H3K9me into the inserted marker genes occurs via an RNAi-dependent mechanism [14], [15]. While H3K9me at *otr1R::ade6^+^* was severely decreased in the white epiclones of *med18Δ* and *med20Δ*, it was substantially retained in the pink epiclones (Figure 2A), indicating that the spreading of H3K9me in the marker genes was variegated in the Mediator mutants. In addition, introduction of, *dcr1Δ* to *med18Δ* cells abolished a variegated phenotype (Figure 3A). These data confirm that the loss of Med18/Mediator results in the variegation of RNAi-dependent heterochromatin spreading. In other words, Med18/Mediator is also involved in the RNAi-dependent heterochromatin pathway.

**Figure 4:**
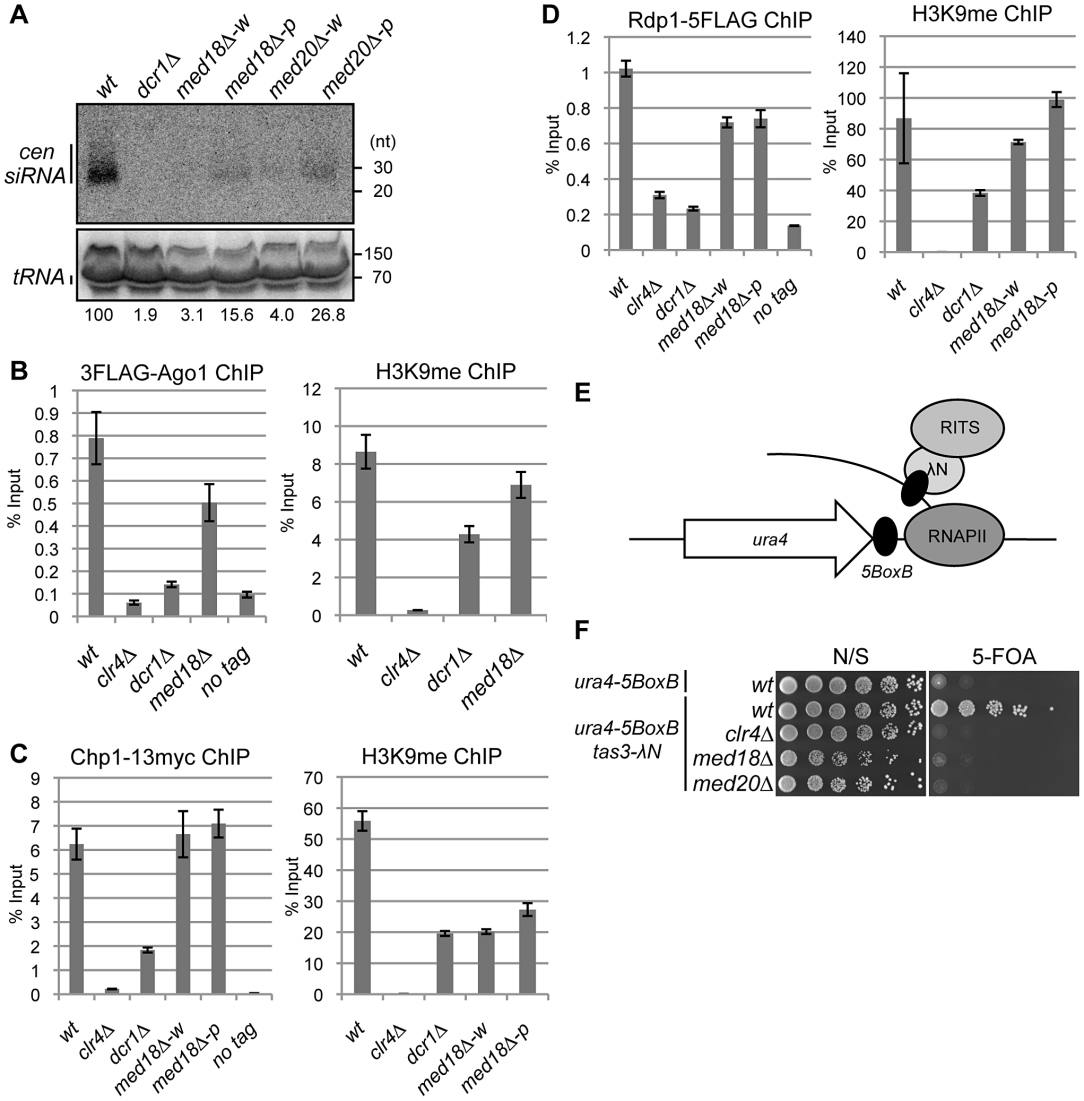
Mediator is required for siRNA formation at the pericentromeric repeats. (A) Northern analysis of siRNA isolated from the indicated strains using oligonucleotide probes specific for *dg* and *dh* centromeric repeats. tRNA was used as a loading control. (B) ChIP analysis of 3Flag-Ago1 and H3K9me at *dh* repeats relative to input WCE in the indicated strains. Error bars show the standard error of the mean (n = 3). (C) ChIP analysis of Chp1-13myc and H3K9me at *dh* repeats relative to input WCE in the indicated strains. Error bars show the standard error of the mean (n = 3). (D) ChIP analysis of Rdp1- 5Flag and H3K9me at *dh* repeats relative to input WCE in the indicated strains. Error bars show the standard error of the mean (n = 3). (E) Schematic representation of artificial heterochromatin formation by RNA-induced transcriptional silencing (RITS) tethering to the *ura4* mRNA. In this system, RITS was tethered artificially to *ura4* RNA via binding of the λN protein fused to Tas3 (a subunit of RITS) to its recognition sequence, BoxB, five copies of which are inserted into the 3′ UTR of the *ura4* mRNA. This induced siRNA generation and subsequent heterochromatin formation at the *ura4* locus in an RNAi-dependent manner. (F) Gene silencing of *ura4* via tethering of RITS. Serial dilution of strains harboring the RITS-tethering system (*ura4-5boxB*, *tas3λN*) or *ura4-5boxB* alone were spotted onto N/S and 5-FOA media for evaluating the silencing state of *ura4* gene. A strain harboring *clr4Δ* was also included as a control.

To examine the involvement of Mediator in RNAi, siRNA derived from *dg* and *dh* repeats was analyzed in the Mediator mutants. siRNAs corresponding to the pericentromeric repeats were not detected in *dcr1Δ* cells (Figure 4A). In the white epiclones of the Mediator mutants, a marginal amount of siRNA from the *dg* and *dh* repeats was detected (Figure 4A and Figure S5A, B). The marginal amount of siRNA was diminished by introduction of *dcr1Δ* (Figure S5B), showing that the siRNA observed in *med18Δ* cells are produced through RNAi pathway. In the pink epiclones, reduced but significant amounts of siRNAs (approximately 10–50% of that of wild-type cells) were detectable. Note that the amount was varied because of the state of variegation. Since the structure of heterochromatin (H3K9me and Swi6) at the repeats was substantially maintained in both white epiclones (Figure 2A, B) and the maintenance was dependent upon RNAi-pathway as shown above (Figure 3C), the small amount of siRNA synthesized in the white epiclones appears to be sufficient to maintain heterochromatin structure at the repeats. A similar reduction of siRNA was observed in the white and pink epiclones of *med8-K9* cells and *med31-H1* cells (Figure S5). Note that when siRNA derived from *dg* and *dh* repeats was analyzed separately, each siRNA was found to be reduced in *med18Δ* cells (Figure S5). These data indicate that Mediator is required for siRNA generation at pericentromeric heterochromatin and the defect of the MHD causes variegation of the spreading of H3K9me into the marker genes.

### Mediator promotes efficient siRNA production from RITS-bound ncRNA

Since RNAi machinery localizes on heterochromatin for processing of ncRNA into siRNA [3], [6], [12], [32], the requirement of Med18 for the recruitment of RNAi factors to heterochromatin was investigated. Binding of the components of the RITS complex (3Flag-Ago1 and Chp1-13myc) and of RDRC (Rdp1-5Flag) to pericentromeric repeats was examined by ChIP assay (Figure 4 B–D). As reported, 3Flag-Ago1 bound to *dh* repeats in a heterochromatin- and/or RNAi-dependent manner [3], [12], [32], as evidenced by the finding that the binding of Ago1 was reduced to a level comparable to that of the no-Flag-tag control in *clr4Δ* and *dcr1Δ* cells (Figure 4B left panel). By contrast, a substantial amount of 3Flag-Ago1 was retained in *med18Δ* cells that formed a mixture of pink and white epiclones (Figure 4B left panel). Binding of Chp1-13myc to *dh* repeats was abolished by deletion of *clr4*, while reduced but significant Chp1-13myc localization was observed at the *dh* repeats in *dcr1Δ* cells, representing the binding of the chromo-domain of Chp1 to H3K9me that was retained at the pericentromeric repeats in these cells (Figure 4C, right panel). By contrast, the binding of Chp1-13myc was not affected by the deletion of *med18*. Even in white epiclones, in which H3K9me is reduced to the same level as in *dcr1Δ* cells (Figure 4C, right panel), Chp1-13myc binds to *dh* repeats at the same level as in wild-type cells (Figure 4C, left panel). Association of Rdp1-5Flag in each mutant was similar to that of 3Flag-Ago1 in that it was almost abolished in *clr4Δ* and *dcr1Δ* cells, but significantly retained in both *med18Δ-w* and *med18Δ-p* cells (Figure 4D, left panel). All together, the RITS complex and RDRC associated with heterochromatin even in *med18Δ-w* cells, probably because the small amount of siRNA synthesized in *med18Δ* cells was sufficient for the association of RITS with heterochromatin. Together with the data on the accumulation of ncRNA and reduction of siRNA in Mediator mutants, these data indicate that Mediator is not required for the association of the RITS complex and RDRC to heterochromatin but is required for efficient siRNA production from the ncRNA by heterochromatin-bound RNAi machinery.

It has been previously reported that the tethering RITS to *ura4* RNA induces RNAi- and heterochromatin-dependent gene silencing of the *ura4* gene, indicating that binding of the RITS complex to ncRNA is a key step in the RNAi-directed formation of heterochromatin by inducing H3K9 methylation and conversion of ncRNA to siRNA [33]. Tethering of RITS is achieved by the fusion of Tas3, a subunit of the RITS complex, to the λN protein, which binds to the 5BoxB sequence inserted at the 3′ UTR region of *ura4* RNA (Figure 4E). To determine whether Med18 or Med20 is required for Tas3-λN-induced silencing of the *ura4-5boxB* gene, the effect of the deletion of these subunits on silencing induced by artificial tethering of the RITS complex was examined. Disruption of *med18* or *med20* resulted in the loss of *ura4-5BoxB* silencing (Figure 4F), similar to the effect of *clr4* disruption. This result showed that Med18 and Med20 are required for Tas3-λN-induced silencing of the *ura4-5BoxB* locus and that Mediator plays a role in the step following the binding of the RITS complex to target RNA.

### Mediator is required for efficient transcription in heterochromatin

**Figure 5:**
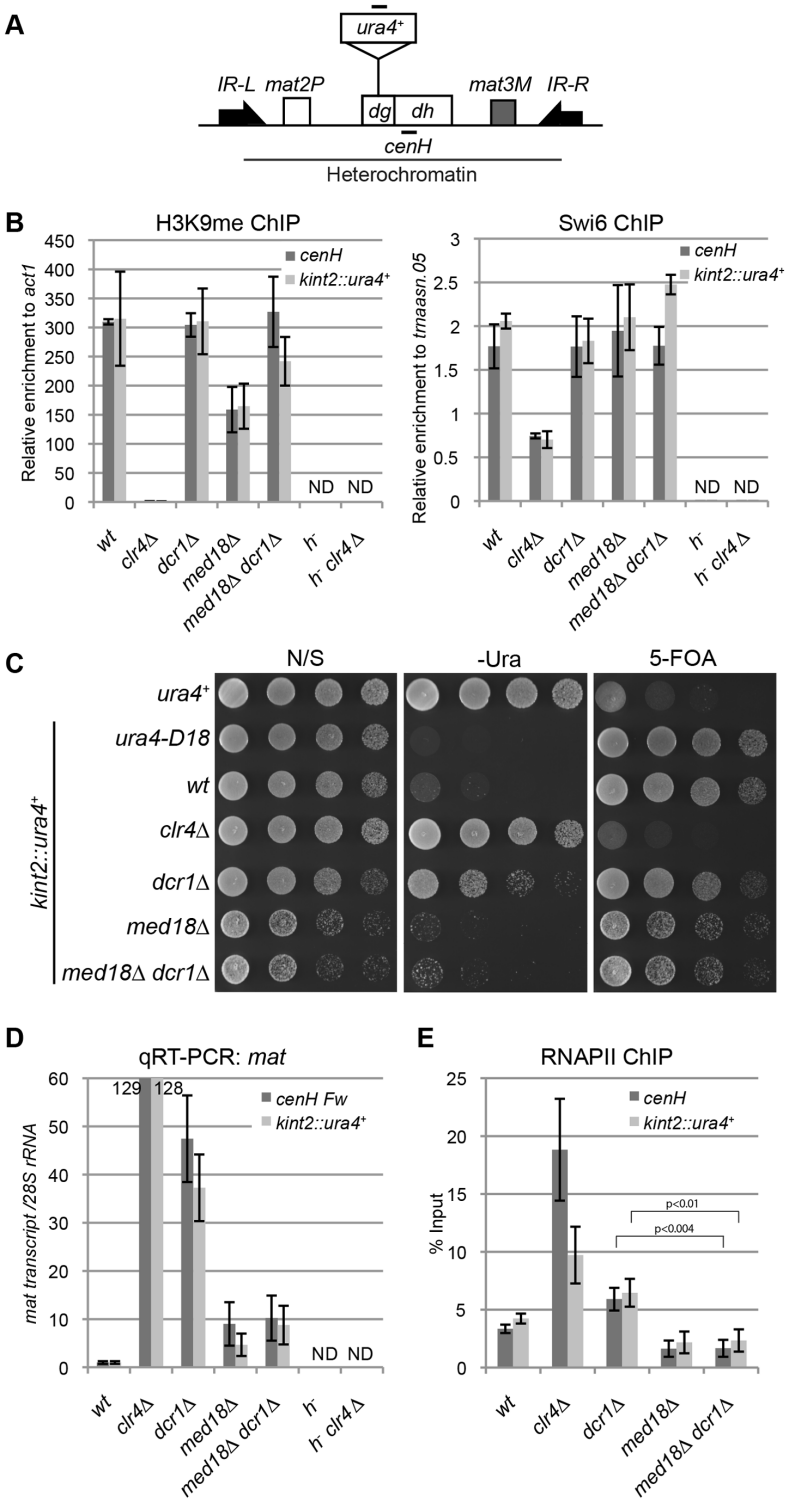
Mediator is required for transcriptional activation in heterochromatin. (A) Schematic of the fission yeast mating-type locus. Location of the *ura4* reporter inserted within the *cenH* is shown (*kint2::ura4^+^*). Black bars indicate the location of primers or probes used for ChIP, RT-PCR and northern analysis. (B) ChIP analysis of H3K9me and Swi6 at *cenH dh* repeats or *kint2::ura4^+^*, each relative to *act1* or *trnaasn.05*. Error bars show the standard error of the mean (n = 3). (C) Silencing assay at the mating-type locus. The results of serial dilutions of the indicated strains spotted onto N/S, 5-FOA media and medium without uracil (-URA) for the silencing of *ura4* are shown. Note that PMGS (PM medium containing L-glutamic acid as a nitrogen source instead of ammonium chloride) plates were used as N/S plates. (D) Quantitative RT-PCR analysis of *cenH dh* repeats or *kint2::ura4^+^* forward transcript levels relative to a control *SPRRNA.48*, whose levels were normalized to that of the wild-type in the indicated strains. Error bars show the standard error of the mean (n = 3). (E) ChIP analysis of RNAPII at *cenH dh* repeats or *kint2::ura4^+^* relative to *SPRRNA.48*. Error bars show the standard error of the mean (n = 3). P values were determined using a two-sided Student's t-test. Note that in Figures B, D and E, *h^−^* strains (*h^−^, h^−^clr4Δ*), which do not have *cenH* sequence, were included to show the primers used in the experiments only detect *cenH* and do not detect the pericentromeric repeats.

Since Mediator regulates general transcription in euchromatin, it is possible that it also regulates the transcription of heterochromatic non-coding RNA. Indeed, recent reports suggest a negative role of MHD subunits (Med18 and Med20) in heterochromatic transcription, based on the observation of an increase in the transcription of pericentromeric ncRNA in Mediator mutants [34], [35]. However, it is difficult to state conclusively whether the observed increase is due to the direct effects of the absence of Mediator because it is also possible that the deletion of Mediator subunits causes disruption of the heterochromatin, which secondarily induces an increase in transcription. To avoid this dilemma, heterochromatin at the mating-type locus was selected for examination (Figure 5A) because the RNAi-dependent pathway is dispensable for the maintenance of heterochromatin here due to the existence of another pathway mediated by the DNA-binding proteins Atf1and Pcr1 [20]. Thus, mutation of Mediator would not be expected to affect the mating-type locus heterochromatin, making it possible to directly measure the effect of the mutation on transcription activity in heterochromatin.

First, a ChIP assay was performed for H3K9me and Swi6 to examine heterochromatin structure at the mating-type locus in various mutants using specific primers for the *cenH* sequence (Figure 5B). As expected, high levels of H3K9me2 and Swi6 were maintained at the *cenH* sequence at the mating-type locus and the inserted *ura4^+^* gene (*kint2::ura4^+^*) in *dcr1Δ* and *dcr1Δ med18Δ* mutants. In *med18Δ* cells, the level of H3K9me was decreased to half of that of wild-type cells for an unknown reason, but the level of Swi6, which is essential for transcriptional gene silencing in heterochromatin, was maintained, consistent with the observation that silencing at this locus was not affected by *med18Δ* (Figure 5B). Hence, the Atf1/Pcr1-dependent pathway probably retains heterochromatin structure and silencing without Med18 function.

Next, the effect of deletion of *dcr1* and *med18* on the silencing of *kint2::ura4^+^* was examined (Figure 5C). While the wild-type strain was able to grow on a 5-FOA-containing plate but not on a uracil-lacking (-Ura) plate, the *clr4Δ* strain was hypersensitive to 5-FOA but grew well on an –Ura plate (Figure 5C), showing that *kint2::ura4^+^* was silenced and expressed, respectively, in each strain. By contrast, *dcr1Δ* cells, like the wild-type cells, hardly grew on 5-FOA containing media, while some cells were able to grow on an –Ura plate, suggesting that silencing is only weakly compromised in *dcr1Δ* cells. However, *med18Δ* cells showed a phenotype similar to that of wild-type cells, showing no silencing defect at the mating-type locus. Introduction of *med18Δ* to *dcr1Δ* cells suppressed the silencing defect detected on the –Ura plate. RT-PCR analysis of transcripts from *cenH* and *kint2::ura4^+^* was consistent with the silencing assay; more than 100-fold, approximately 40-fold, and approximately 10-fold accumulation of transcripts from *cenH* and *kint2::ura4* were observed in *clr4Δ*, *dcr1Δ* and *med18Δ* cells, respectively (Figure 5D). In addition, introduction of *med18Δ* into *dcr1Δ* cells caused a decrease in transcripts to a level similar to that of *med18Δ* cells.

The accumulation of RNA in *dcr1Δ* cells and *med18Δ* cells could be explained by defects in RNA degradation by RNAi and/or the exosome (Noma et al., 2004, Buhler et al., 2007), or by an increase in heterochromatic transcription. To examine the latter possibility, localization of RNAPII at *cenH* and *kint2::ura4^+^* was examined by ChIP assay (Figure 5E). Unexpectedly, RNAPII was significantly increased in *dcr1Δ* cells at both loci in spite of the maintenance of heterochromatin in this strain, suggesting that Dcr1 negatively regulates heterochromatin transcription. Note that RNAi machinery has been shown to interact with RNAPII and modulate transcription in other organisms [36], [37]. Thus, an increase in transcription and prevention of processing of RNA into siRNA could cause the observed accumulation of transcripts in *dcr1Δ* cells (Figure 5D). By contrast, the level of RNAPII in *med18Δ* cells was comparable to that of wild-type cells (Figure 5E). Similar results of RNAPII localization were obtained with ChIP assay using the antibody against RNAPII-C-terminal repeats phosphorylated at the second serine, which represents elongating RNAPII (Figure S6). These indicated that Med18 does not repress transcription in heterochromatin. The approximately 10-fold accumulation of *cenH* and *kint2::ura4^+^* RNA observed in *med18Δ* cells might be due to a defect in exosome-dependent degradation of RNA [16], which would indicate that significant transcription took place in the absence of Med18. Importantly, introduction of *med18Δ* into *dcr1Δ* cells caused a decrease in RNAPII to the level of wild-type cells, suggesting that Mediator is required for efficient transcription in heterochromatin in *dcr1Δ* cells.

To analyze the role of Med18 on the transcription in the absence of heterochromatin, we compared RNAPII occupancy at centromeric repeats of *dcr1Δ rrp6Δ* cells with those of *dcr1Δ rrp6Δ med18Δ*cells (Figure S7). Note that both strains showed similarly low levels of H3K9me at *dh* repeats (Figure 3C). Introduction of *med18Δ* caused the moderate increase of RNAPII. This suggested that Med18 negatively regulates transcription in the compromised heterochromatin.

From these data, we suggest that in the fully assembled heterochromatin, Med18/Mediator does not negatively regulate pericentromeric transcription; rather, it might be required for efficient transcription in heterochromatin.

### Effect of Mediator disruption on euchromatic genes

**Figure 6:**
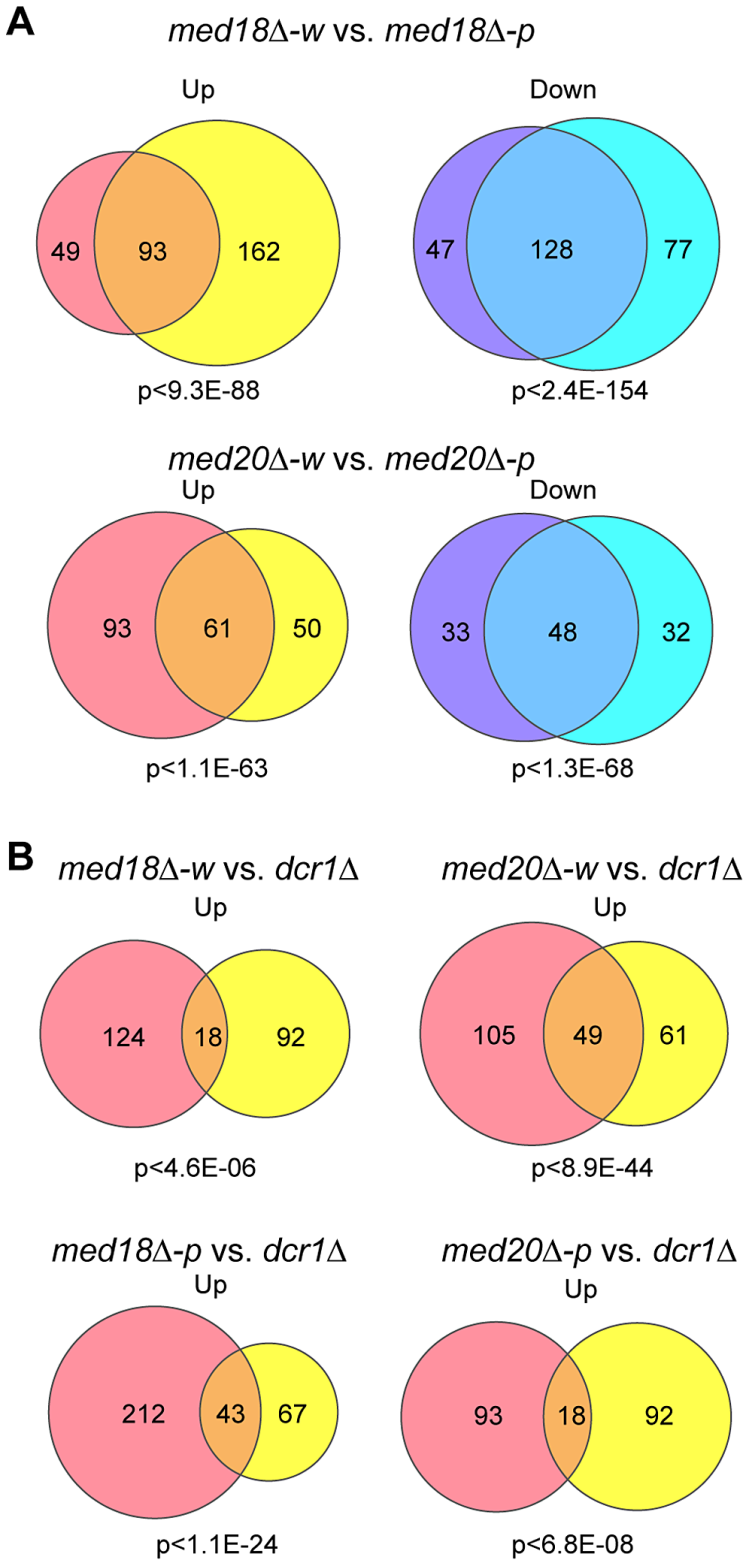
Effect of Mediator disruption on euchromatic genes. Venn diagram showing the number of transcripts whose expression levels are increased (up) or decreased (down) >1.5-fold in mutants compared to the wild-type. P-values were calculated using Fisher's exact test. (A) Transcripts of *med18Δ-w* (left circles) vs. *med18Δ-p* (right circles) mutants (top) and *med20Δ-w* (left circles) vs. *med20Δ-p* (right circles) mutants (bottom). (B) Transcripts of *med18Δ-w* (left circles) vs. *dcr1Δ* (right circles) mutants (upper left), *med20Δ-w* (left circles) vs. *dcr1Δ* (right circles) mutants (upper right), *med18Δ-p* (left circles) vs. *dcr1Δ* (right circles) mutants (lower left), and *med20Δ-p* (left circles) vs. *dcr1Δ* (right circles) mutants (lower right).

The effect of *med18Δ* and *med20Δ* on euchromatic gene expression was further examined using microarray. Pink and white epiclones of *med18Δ* and *med20Δ* cells were separated and the expression pattern of each epiclone was compared. Analysis of the genes that showed ≥1.5-fold increase (Up) or decrease (Down) in expression between the epiclones revealed that a common set of genes were affected in both *med18Δ* cells and *med20Δ* cells, irrespective of the state of heterochromatic silencing (Figure 6A). Therefore, a clear difference between the white and pink epiclones was observed at pericentromeric ncRNA expression. Indeed, in the white epiclones, no euchromatic genes showed stronger induction than centromeric ncRNA; the most strongly increased euchromatic gene showed an approximately 14-fold and 26-fold increase in *med18Δ-w* and *med20Δ-w* cells, respectively, which is much weaker than the increase in centromeric ncRNA (which increased by more than 100-fold). Comparison of *med18Δ-w* and *med20Δ-w*, or *med18Δ-p* and *med20Δ-p*, showed that both subunits shared a common set of targets (Figure S8). This is consistent with the fact that both Med18 and Med20 formes heterodimers submodule in the MHD.

When the expression pattern in the white epiclones of the Mediator mutants (*med18Δ-w* and *med20Δ-w*) was compared with that in *dcr1Δ* cells, it was evident that the expression of a common set of genes was upregulated (Figure 6B, upper panels). Similar sharing of target genes was observed between the pink epiclones of the Mediator mutants (*med18Δ-p* and *med20Δ-p*) and *dcr1Δ* cells (Figure 6B, lower panel). Interestingly, gene ontology analysis showed significant enrichment of terms pertaining to stress responses in the shared target genes. For example, the top GO terms included “cellular response to stimulus” (GO: 0033554, *med18Δ-w* vs. *dcr1Δ P* = 5.35×10^−3^, *med18Δ-p* vs. *dcr1Δ P* = 2.2×10^−6^, *med20Δ-w* vs. *dcr1Δ P* = 2.19×10^−6^, *med20Δ-p* vs. *dcr1Δ P* = 1.27×10^−4^). These results suggest that some euchromatic genes, including stress response genes, are repressed by the RNAi/Mediator system, which may function via a mechanism partly similar to that of RNAi-mediated heterochromatin.

## Discussion

**Figure 7:**
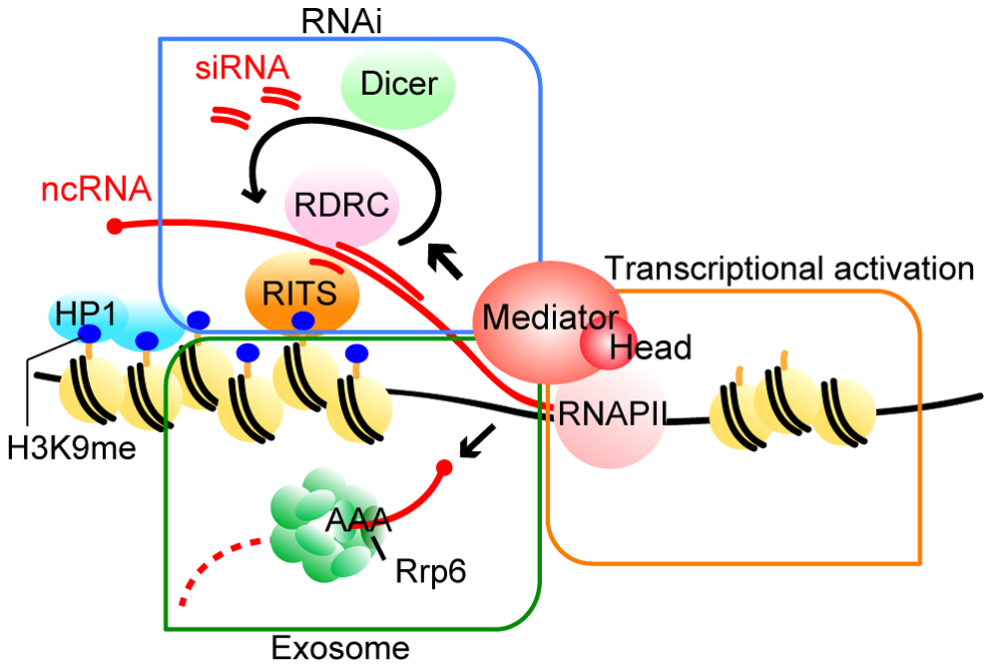
Model of the functions of Mediator in pericentromeric heterochromatin assembly in fission yeast. Mediator localizes to the pericentromeres to recruit RNAPII for efficient transcription. Furthermore, Mediator regulates processing of transcribed ncRNA by RNAi machineries, which directs heterochromatin formation. In addition, Mediator is also required for Rrp6-dependent heterochromatin formation. For details, see Discussion.

In this study, we showed that the specific subunits of Mediator, Med18, Med20, Med8 and Med31 were involved in pericentromeric heterochromatin formation. The Med18-Med20 heterodimer is a component of the head domain of Mediator [26]. Because the *med8-K9* mutation causes truncation of the C-terminal domain that interacts with the Med18/Med20 heterodimer (Figure S1B), it resulted in the loss of the heterodimer. Importantly, Med31 belongs to the middle domain but is located close to the head domain [38], suggesting that the head domain does not function alone in heterochromatin formation, but rather as a part of Mediator. Therefore, we suggest that Mediator specifically plays multiple roles in the formation of pericentromeric heterochromatin via the MHD (Fig. 7).

Two distinct mechanisms, the RNAi-dependent and Rrp6-dependent pathways, function in heterochromatin formation at pericentromeric repeats, while the spreading of H3K9me onto marker genes mainly depends on the RNAi pathway [14], [15]. We found that at the pericentromeric heterochromatin, in the absence of MHD, the Rrp6-dependent pathway is compromised and H3K9me is largely maintained by the RNAi-dependent pathway (Fig. 3). The finding that the amount of siRNA produced in the white epiclones of MHD mutants decreased to 3–20% of that in wild-type cells (Fig. 4A and Figure S5) indicates that only a small amount of siRNA is necessary to maintain heterochromatin at the pericentromeric repeats. The remaining H3K9me at the repeats in MHD mutants spreads onto the marker genes by an RNAi-dependent mechanism. This process was also compromised by the decrease of siRNA caused by the absence of MHD, resulting in variegation of the level of H3K9me at the marker genes, which ultimately caused the appearance of white and pink epiclones. The spreading process appears to require more efficient siRNA production than the maintenance of heterochromatin at the pericentromeric repeats because *med18Δ-p* cells that produced more siRNA showed more efficient spreading of H3K9me and silencing of marker genes than *med18Δ-w* cells. In contrast, *dcr1Δ* cells did not show the variegation of silencing because of loss of siRNA production. It is noteworthy that the variegated phenotype was metastable, which suggests that once heterochromatin was spread into the marker gene, it could be maintained in an MHD-independent manner, probably through the small amount of siRNA produced in MHD mutants.

Many processes involved in RNA processing, such as RNA splicing and RNA transport, are coupled to transcription by RNAPII. siRNA production is also coupled with RNAPII-dependent transcription [10]. Our results showed that the mutation of MHD resulted in a large decrease in siRNA. By contrast, *rrp6Δ* cells, which have levels of H3K9me and Swi6 at the pericentromeric repeats similar to *med18Δ* cells (Fig. 3), produced the same amount of siRNA as wild-type cells [16]. These results indicate that MHD is somehow involved in siRNA production after transcription of ncRNA. MHD is localized at the transcribed region in pericentromeric repeats (Figure S4; [34], [35] and is required for transcription in heterochromatin, suggesting that it directly functions in the coupling of transcription of heterochromatic ncRNA by RNAPII and processing of the siRNA by RNAi machinery. Retention of RNAi factors at the pericentromeric repeats in *med18Δ* cells and the requirement for MHD in heterochromatin formation via the artificial tethering of the RITS complex to RNA suggest that MHD functions after RITS associates with heterochromatic repeats and/or target RNA. Recently, we showed that RNAi factors are assembled into an siRNA amplification compartment that includes transcriptionally active heterochromatin [39]. Thus, MHD might be involved in the formation of this compartment. Although we were not able to detect a stable interaction between MHD components and RNAi factors, such as Ago1, by co-immunoprecipitation experiments (data not shown), it is still possible that MHD recruits factors required for siRNA generation to transcriptionally active heterochromatin through direct or indirect interactions. In any case, further experiments are necessary to clarify the molecular function of Mediator in the RNAi pathway.

In contrast to the RNAi pathway, little is known about the Rrp6-dependent heterochromatin formation pathway. As deletion of *rrp6* marginally affects H3K9me and silencing at the inserted marker genes [16], [18], the Rrp6-dependent pathway mainly functions at the pericentromeric repeats. Our genetic experiments showed that *rrp6* and *med18* were epistatic in the formation of pericentromeric heterochromatin (Fig. 3), indicating that MHD functions in Rrp6-dependent heterochromatin formation. Rrp6 is an exonuclease that is a subunit of the nuclear exosome involved in RNA-quality control [40]. A functional relationship between Mediator and the nuclear exosome has not been reported. We found that Rrp6 associates with both heterochromatin (*dh*) as well as euchromatin (*act1* and *fbp1*) and deletion of *med18* did not affect the localization, suggesting Mediator acts in a step after the association of Rrp6/exsosome with chromatin. This is analogous to the function of Mediator in the RNAi-dependent pathway; MHD functions after recruitment of RITS complex and RDRC to chromatin. Considering the co-transcriptional nature of RNA-quality control [41], [42] and recruitment of RNA-splicing factors to transcripts by Mediator [29], we speculate that MHD plays a role in the co-transcriptional function of chromatin-associated Rrp6 and/or other co-factors to promote heterochromatin formation. Alternatively, given that the RNAi-independent heterochromatin nucleation pathway and Mediator functionally interact with RNAPII processivity factors [18], [30], Mediator may promote Rrp6-dependent heterochromatin formation by affecting elongation by RNAPII through interaction with these processivity factors. It is also possible that the same mechanism is also involved in RNAi-dependent heterochromatin formation through MHD.

Recently, two reports showed that MHD was important for heterochromatin formation at pericentromeres [34], [35]. However, there are several discrepancies between their data and ours. Firstly, the decrease in H3K9me and Swi6 in *med20Δ* cells was much more severe than that in ours. Secondly, Carlsten et al. claimed that siRNA from the *dh* repeat in *med20Δ* cells was diminished but that siRNA from the *dg* repeats was comparable to that in wild-type cells. Thirdly, both papers assert that Mediator negatively regulates heterochromatic transcription. The first two discrepancies could be caused by the variegated phenotype of MHD mutants. If this variegated phenotype was overlooked or disregarded, the results would be affected by which epiclones were used in the experiments. In addition, since the amount of H3K9me/Swi6 varies depending on the position in the repeats, the discrepancies between their results and ours might reflect a difference in the sites used for ChIP analysis. The third discrepancy could be explained by the use of pericentromeric transcription for their analysis. As described in the Results section, it is hard to argue definitively for the direct influence of MHD mutants on transcription in pericentromeric heterochromatin because it is difficult to determine whether the observed increase is due to the direct effect of depletion of MHD or a secondary effect resulting from the disruption of heterochromatin. Our data using the mating-type locus heterochromatin showed that disruption of Mediator did not cause increased transcription in heterochromatin, rather it caused a decrease in transcription enhanced by deletion of *dcr1* (Fig. 6). Interestingly, when heterochromatin was compromised, Mediator appears to negatively regulate transcription, which might also explain the discrepancy.

Recently, Dcr1 was shown to repress a set of genes, including stress response genes, through the degradation of target RNA [43]. We identified a similar set of euchromatic genes that were up-regulated in *med20Δ* and *dcr1Δ* cells, suggesting that MHD functions in co-transcriptional degradation of euchromatic RNAs in collaboration with Dcr1. Note that previous transcriptome analysis of the mediator mutants also showed that a similar set of genes were up regulated in *med18Δ* and *med20Δ* cells but not in *med12Δ* cells, supporting the collaborative function of MHD and Dcr1 [44]. In addition, Rrp6-dependent heterochromatin formation was observed at several meiotic genes [45]. Moreover, the exosome and RNAi are shown to regulate a set of genes, including retrotransposons and developmental genes [46]. Therefore, it is also possible that Mediator functions at these loci to silence genes by regulating both RNAi and exosomal machineries.

Emerging evidence shows that Mediator works as a platform for various factors that function in transcription and RNA processing, using a distinct subunit for particular interactions with the factors [29], [30]. Our results further extend the range of Mediator function to include regulation of higher-order chromatin structure in the genome. It is now widely accepted that RNAPII transcribes almost all of the genome. Mediator might not only mediate transcription factors and RNAPII at each gene, but also mediate RNAPII and genome-wide regulation of higher-order chromatin structure.

## Materials and Methods

### Strains and culture media

The *S. pombe* strains used in this study are described in Table S1. The media and genetic methods used in the study were essentially as described previously [47]. Yeast cells were cultured in YES at 30°C. For deletion or epitope-tagging of the target genes, the PCR-based module method [48] was used.

### Silencing assays

Silencing assays were conducted from overnight unsaturated cultures grown in 10 ml YES. A 5-fold dilution series of cells was spotted on N/S plates (YES in all spot figures, except Figure 5B), 5-FOA plates (N/S plates with the addition of 1 g/l 5-fluoroorotic acid), and Low Ade plates (N/S plates including limited amount of adenine). The plates were then incubated at 30°C for 3 days.

### Chromatin immunoprecipitation (ChIP) analysis

ChIP was performed as described previously [39] with the following changes: crosslinking with formaldehyde was performed for 30 min and digestion with Proteinase K was carried out for 1 h at 42°C. The following antibodies were used: anti-H3-K9-me2 monoclonal (a gift from T. Urano, Shimane University), anti-Swi6 (produced in-house), anti-RNA polymerase II (8WG16, Abcam), anti-FLAG (M2, Sigma), or anti-myc (4A6, Millipore). Primer sequences are shown in Table S2.

### RNA analysis

Total RNA was isolated from logarithmically-growing *S. pombe* (in YES media) using the hot phenol method [49]. For northern blotting of centromeric and mat RNA, 50 µg of total RNA was electrophoresed on a 1% agarose gel containing 1× MOPS and 1% formaldehyde. RNA was transferred to positively-charged nylon membranes (Amersham Biosciences) in 10× SSC by standard capillary blotting. Following UV crosslinking of the RNA to the nylon filter, prehybridization and hybridization were carried out at 42°C in UltraHyb-Oligo buffer (Ambion). For hybridization, 50 pmol oligos were end-labeled with [γ^32^P]dATP (3000 Ci/mmol) using T4 Polynucleotide Kinase (TOYOBO). After hybridization for 24 h, membranes were washed four times in 2× SSC/0.1% SDS for 10 min at 42°C before exposure to an imaging plate for 1–2 days. For re-probing, probes on the membrane were stripped by boiling in 200 ml of 0.5× SSC/0.1% with shaking. Detection of siRNA was performed as described previously [39]. Oligonucleotides used as probes are shown in Table S2.

### RT-PCR

For RT-PCR analysis, total RNA was cleaned up and treated with Recombinant DNase I (RNase-free) (TaKaRa) according to the manufacturer's instructions. RT-PCR was performed using PrimeScript Reverse Transcriptase (TaKaRa) according to the manufacturer's instructions. Primer sequences are shown in Table S2.

### qPCR

qPCR was performed using SYBR premix Ex-Taq (TaKaRa) and the Thermal Cycler Dice Real time system TP800 (TaKaRa). Primer sequences are shown in Table S2.

### ChIP-qPCR and RT-PCR using synchronized cdc25-22 cells

*cdc25-22* cells were grown at 25°C to a concentration of 2×10^6^ cells/ml and then shifted to 36°C for 4 hr and 15 min to stop the cell cycle at the G2/M phase. Samples for ChIP assay were collected every 30 min for 300 min after shifting the cells back to 25°C to release cell cycle block. ChIP assay was performed as described in the Experimental Procedures. To prepare RNA for RT-PCR, the input fractions of ChIP were adjusted to 0.25% SDS and 0.25 mg/ml proteinase K and incubated for 45 min at 45°C and then at 65°C for more than 4 hours to reverse crosslinking. Samples were extracted once with phenol-chloroform. After ethanol precipitation, the samples were resuspended in a suitable volume of DEPC-treated distilled water. RT-PCR was performed as described in the Experimental Procedures.

### Microarray analysis of Mediator mutants

Microarray analysis for gene expression was performed as described previously [50] using FY2002 as a parental strain. White and pink epiclones of *med18Δ* and *med20Δ* were analyzed separately. The sequences of the probes and original data from the microarray experiments were deposited to GEO (http://www.ncbi.nlm.nih.gov/geo) with accession number GSE43543.

### Methods in the supplemental information

Methods used in the supplemental information are described in Text S1.

## Supporting Information

Figure S1 Mediator is required for heterochromatin silencing at the pericentromere. (A) Silencing assay at the pericentromere. Shown are the results of serial dilutions of the indicated strains spotted onto non-selective media (N/S), medium without uracil (-Ura), medium with 5-fluoroorotic acid (5-FOA), and medium without adenine (-Ade) to assay *ura4^+^* and *ade6^+^*expression. Note that PMGS (EMMS-NH_4_Cl (nitrogen), +L-glutamic acid, monosodium, as nitrogen) plates were used as N/S plates. (B) Schematic of Med8-K9 and Med31-H1 proteins. *med8-K9* contains a point mutation (C540T) causing C-terminal truncation of the Med8 protein (Q135*) (top). The C-terminal residues 176–200 of Med8 (Med8C) are the predicted interface for interaction with Med18 [26], [51]. *med31-H1* contains a point mutation (T230A) causing C-terminal truncation of the Med31 protein (L77*) (bottom). Residues 22–77 of Med31 are the predicted interface for interaction with Med7, a core Mediator subunit belonged to the middle domain. (C) Silencing assay at the pericentromere. Shown are the results of serial dilutions of the indicated strains spotted onto non-selective media (N/S), medium with 5-FOA, and medium with a limited amount of adenine (Low Ade) to assay for the presence of *imr1L::ura4^+^* and *otr1R::ade6^+^*.
(DOC)

Figure S2 Conversion rates of white/pink epiclones. Stability assay of variegation phenotypes. Conversion rates (percentage of cells that convert the epigenetic state per generation) were measured using indicated strains. *w* indicates the white epiclones and *p* indicates the pink epiclones. Cells were grown for several generations. The rates were measured as described in supplemental experimental procedures.
(DOC)

Figure S3 Mediator mutations cause accumulation of centromeric RNA. (A) Schematic of fission yeast centromere 1. Locations of the *ura4^+^* and *ade6^+^* reporters inserted within the pericentromeric region are shown (*imr1L::ura4^+^* and *otr1R::ade6^+^*). Black bars indicate the location of probes used for northern analysis. (B) Northern analysis of pericentromeric transcripts in wild-type and mutant cells. Analysis was performed using oligonucleotide probes against *dh* and *dg* forward (Fw) and reverse (Rv) strands, *imr1L::ura4^+^* and *otr1R::ade6^+^* forward strand transcripts. rRNA and tRNA were used as loading controls.
(DOC)

Figure S4 Med20 localizes the pericentromeric heterochromatin with RNAPII in a cell cycle-dependent manner. (A, B) ChIP-qPCR analyses for RNAPII and Med20-5FLAG were performed every 30 minutes after release from G2/M block by *cdc25-22* mutation (see Supplemental Experimental Procedures). Enrichment at *dh* repeats relative to the *adh1* promoter region (*adh1 pro*) is shown. Error bars show the standard error of the mean (n = 3). (C) Septation index (percentage of cells with division septum) was measured to monitor cell cycle progression after release. In a block and release experiment, the peak of septation (90 to 120 min) indicates S phase. (D) RT-PCR of *dh* transcripts was performed as described in A and B. Transcripts derived from constitutively expressed *act1* served as controls. “No RT” indicates that no reverse transcriptase was added in the reaction. Numbers under the panels of *dh* and *act1* indicate the increase in transcript relative to the values at 0 min (*dh*) and 60 min (*act1*), respectively.
(TIF)

Figure S5 Northern analysis of siRNA in the Mediator mutants *med8-K9* and *med31-H1*. Analyses were performed with RNA isolated from the indicated strains (panel A: single deletion mutants, panel B: single and double deletion mutants of *dcr1* and *med18*) using oligonucleotide probes specific for *dg/dh* centromeric repeats. tRNA was used as a loading control.
(DOC)

Figure S6 Mediator is required for transcriptional activation in heterochromatin. ChIP analysis of RNAPII at *cenH dh* repeats or *kint2::ura4^+^* relative to input WCE in the indicated strains. Anti-RNA polymerase II C-terminal domain (CTD) serine 2 phosphorylation was used. Error bars represent the standard error of the mean (n = 3). P values were determined using a two-sided Student's t-test.
(TIF)

Figure S7 Mediator negatively regulates RNAPII in the compromised heterochromatin. ChIP analysis of RNAPII at *dh* repeats relative to the gene free region in the indicated strains. Error bars show the standard error of the mean (n = 3).
(TIF)

Figure S8 Effect of Mediator disruption on euchromatic genes. Venn diagram showing the number of transcripts whose expression levels were increased (up) or decreased (down) >1.5-fold in mutants compared to the wild type. The p-value was calculated using Fisher's exact test. (A) Transcripts of *med18Δ-w* (left circles) vs. *med20Δ-w* (right circles) mutants. (B) Transcripts of *med18Δ-p* (left circles) vs. *med20Δ-p* (right circles) mutants.
(DOC)

Table S1 Strains used in this study.
(DOCX)

Table S2 Primers used in this study.
(DOCX)

Text S1 Supplemental methods.
(DOCX)

## References

[1] Rea S, Eisenhaber F, O'Carroll D, Strahl BD, Sun ZW, et al (2000) Regulation of chromatin structure by site-specific histone H3 methyltransferases. Nature 406: 593–599. 10949293 10.1038/35020506

[2] Nakayama J, Rice JC, Strahl BD, Allis CD, Grewal SI (2001) Role of histone H3 lysine 9 methylation in epigenetic control of heterochromatin assembly. Science 292: 110–113. 11283354 10.1126/science.1060118

[3] Cam HP, Sugiyama T, Chen ES, Chen X, FitzGerald PC, et al (2005) Comprehensive analysis of heterochromatin- and RNAi-mediated epigenetic control of the fission yeast genome. Nat Genet 37: 809–819. 15976807 10.1038/ng1602

[4] Grewal SI, Jia S (2007) Heterochromatin revisited. Nat Rev Genet 8: 35–46. 17173056 10.1038/nrg2008

[5] Shimada A, Murakami Y (2010) Dynamic regulation of heterochromatin function via phosphorylation of HP1-family proteins. Epigenetics 5: 30–33. 20083904 10.4161/epi.5.1.10605

[6] Volpe TA, Kidner C, Hall IM, Teng G, Grewal SI, et al (2002) Regulation of heterochromatic silencing and histone H3 lysine-9 methylation by RNAi. Science 297: 1833–1837. 12193640 10.1126/science.1074973

[7] Motamedi MR, Verdel A, Colmenares SU, Gerber SA, Gygi SP, et al (2004) Two RNAi complexes, RITS and RDRC, physically interact and localize to noncoding centromeric RNAs. Cell 119: 789–802. 15607976 10.1016/j.cell.2004.11.034

[8] Chikashige Y, Kinoshita N, Nakaseko Y, Matsumoto T, Murakami S, et al (1989) Composite motifs and repeat symmetry in S. pombe centromeres: direct analysis by integration of NotI restriction sites. Cell 57: 739–751. 2541922 10.1016/0092-8674(89)90789-7

[9] Djupedal I, Portoso M, Spahr H, Bonilla C, Gustafsson CM, et al (2005) RNA Pol II subunit Rpb7 promotes centromeric transcription and RNAi-directed chromatin silencing. Genes Dev 19: 2301–2306. 16204182 10.1101/gad.344205 PMC1240039

[10] Kato H, Goto DB, Martienssen RA, Urano T, Furukawa K, et al (2005) RNA polymerase II is required for RNAi-dependent heterochromatin assembly. Science 309: 467–469. 15947136 10.1126/science.1114955

[11] Chen ES, Zhang K, Nicolas E, Cam HP, Zofall M, et al (2008) Cell cycle control of centromeric repeat transcription and heterochromatin assembly. Nature 451: 734–737. 18216783 10.1038/nature06561

[12] Noma K, Sugiyama T, Cam H, Verdel A, Zofall M, et al (2004) RITS acts in cis to promote RNA interference-mediated transcriptional and post-transcriptional silencing. Nat Genet 36: 1174–1180. 15475954 10.1038/ng1452

[13] Sugiyama T, Cam H, Verdel A, Moazed D, Grewal SI (2005) RNA-dependent RNA polymerase is an essential component of a self-enforcing loop coupling heterochromatin assembly to siRNA production. Proc Natl Acad Sci U S A 102: 152–157. 15615848 10.1073/pnas.0407641102 PMC544066

[14] Irvine DV, Zaratiegui M, Tolia NH, Goto DB, Chitwood DH, et al (2006) Argonaute slicing is required for heterochromatic silencing and spreading. Science 313: 1134–1137. 16931764 10.1126/science.1128813

[15] Li H, Motamedi MR, Yip CK, Wang Z, Walz T, et al (2009) An alpha motif at Tas3 C terminus mediates RITS cis spreading and promotes heterochromatic gene silencing. Mol Cell 34: 155–167. 19394293 10.1016/j.molcel.2009.02.032 PMC2756231

[16] Bühler M, Haas W, Gygi SP, Moazed D (2007) RNAi-dependent and -independent RNA turnover mechanisms contribute to heterochromatic gene silencing. Cell 129: 707–721. 17512405 10.1016/j.cell.2007.03.038

[17] Bühler M, Spies N, Bartel DP, Moazed D (2008) TRAMP-mediated RNA surveillance prevents spurious entry of RNAs into the Schizosaccharomyces pombe siRNA pathway. Nat Struct Mol Biol 15: 1015–1023. 18776903 10.1038/nsmb.1481 PMC3240669

[18] Reyes-Turcu FE, Zhang K, Zofall M, Chen E, Grewal SI (2011) Defects in RNA quality control factors reveal RNAi-independent nucleation of heterochromatin. Nat Struct Mol Biol 18: 1132–1138. 21892171 10.1038/nsmb.2122 PMC3190054

[19] Grewal SI, Klar AJ (1997) A recombinationally repressed region between mat2 and mat3 loci shares homology to centromeric repeats and regulates directionality of mating-type switching in fission yeast. Genetics 146: 1221–1238. 9258669 10.1093/genetics/146.4.1221 PMC1208070

[20] Jia S, Noma K, Grewal SI (2004) RNAi-independent heterochromatin nucleation by the stress-activated ATF/CREB family proteins. Science 304: 1971–1976. 15218150 10.1126/science.1099035

[21] Kelleher RJ 3rd, Flanagan PM, Kornberg RD (1990) A novel mediator between activator proteins and the RNA polymerase II transcription apparatus. Cell 61: 1209–1215. 2163759 10.1016/0092-8674(90)90685-8

[22] Kim YJ, Bjorklund S, Li Y, Sayre MH, Kornberg RD (1994) A multiprotein mediator of transcriptional activation and its interaction with the C-terminal repeat domain of RNA polymerase II. Cell 77: 599–608. 8187178 10.1016/0092-8674(94)90221-6

[23] Soutourina J, Wydau S, Ambroise Y, Boschiero C, Werner M (2011) Direct interaction of RNA polymerase II and mediator required for transcription in vivo. Science 331: 1451–1454. 21415355 10.1126/science.1200188

[24] Lariviere L, Seizl M, Cramer P (2012) A structural perspective on Mediator function. Curr Opin Cell Biol 24: 305–313. 22341791 10.1016/j.ceb.2012.01.007

[25] Bourbon HM (2008) Comparative genomics supports a deep evolutionary origin for the large, four-module transcriptional mediator complex. Nucleic Acids Res 36: 3993–4008. 18515835 10.1093/nar/gkn349 PMC2475620

[26] Lariviere L, Geiger S, Hoeppner S, Rother S, Strasser K, et al (2006) Structure and TBP binding of the Mediator head subcomplex Med8-Med18-Med20. Nat Struct Mol Biol 13: 895–901. 16964259 10.1038/nsmb1143

[27] Imasaki T, Calero G, Cai G, Tsai KL, Yamada K, et al (2011) Architecture of the Mediator head module. Nature 475: 240–243. 21725323 10.1038/nature10162 PMC4109712

[28] Kim YJ, Zheng B, Yu Y, Won SY, Mo B, et al (2011) The role of Mediator in small and long noncoding RNA production in Arabidopsis thaliana. EMBO J 30: 814–822. 21252857 10.1038/emboj.2011.3 PMC3049218

[29] Huang Y, Li W, Yao X, Lin QJ, Yin JW, et al (2012) Mediator complex regulates alternative mRNA processing via the MED23 subunit. Mol Cell 45: 459–469. 22264826 10.1016/j.molcel.2011.12.022 PMC3288850

[30] Takahashi H, Parmely TJ, Sato S, Tomomori-Sato C, Banks CA, et al (2011) Human mediator subunit MED26 functions as a docking site for transcription elongation factors. Cell 146: 92–104. 21729782 10.1016/j.cell.2011.06.005 PMC3145325

[31] Allshire RC, Nimmo ER, Ekwall K, Javerzat JP, Cranston G (1995) Mutations derepressing silent centromeric domains in fission yeast disrupt chromosome segregation. Genes Dev 9: 218–233. 7851795 10.1101/gad.9.2.218

[32] Verdel A, Jia S, Gerber S, Sugiyama T, Gygi S, et al (2004) RNAi-mediated targeting of heterochromatin by the RITS complex. Science 303: 672–676. 14704433 10.1126/science.1093686 PMC3244756

[33] Bühler M, Verdel A, Moazed D (2006) Tethering RITS to a nascent transcript initiates RNAi- and heterochromatin-dependent gene silencing. Cell 125: 873–886. 16751098 10.1016/j.cell.2006.04.025

[34] Carlsten JO, Szilagyi Z, Liu B, Davila Lopez M, Szaszi E, et al (2012) Mediator Promotes CENP-A Incorporation at Fission Yeast Centromeres. Mol Cell Biol 32: 4035–43. 22851695 10.1128/MCB.00374-12 PMC3457525

[35] Thorsen M, Hansen H, Venturi M, Holmberg S, Thon G (2012) Mediator regulates non-coding RNA transcription at fission yeast centromeres. Epigenetics Chromatin 5: 19. 23171760 10.1186/1756-8935-5-19 PMC3541127

[36] Kavi HH, Birchler JA (2009) Interaction of RNA polymerase II and the small RNA machinery affects heterochromatic silencing in Drosophila. Epigenetics Chromatin 2: 15. 19917092 10.1186/1756-8935-2-15 PMC2785806

[37] Guang S, Bochner AF, Burkhart KB, Burton N, Pavelec DM, et al (2010) Small regulatory RNAs inhibit RNA polymerase II during the elongation phase of transcription. Nature 465: 1097–1101. 20543824 10.1038/nature09095 PMC2892551

[38] Cai G, Imasaki T, Yamada K, Cardelli F, Takagi Y, et al (2010) Mediator head module structure and functional interactions. Nat Struct Mol Biol 17: 273–279. 20154708 10.1038/nsmb.1757 PMC2925518

[39] Kawakami K, Hayashi A, Nakayama J, Murakami Y (2012) A novel RNAi protein, Dsh1, assembles RNAi machinery on chromatin to amplify heterochromatic siRNA. Genes Dev 26: 1811–1824. 22895252 10.1101/gad.190272.112 PMC3426760

[40] Houseley J, LaCava J, Tollervey D (2006) RNA-quality control by the exosome. Nat Rev Mol Cell Biol 7: 529–539. 16829983 10.1038/nrm1964

[41] Huertas P, Aguilera A (2003) Cotranscriptionally formed DNA:RNA hybrids mediate transcription elongation impairment and transcription-associated recombination. Mol Cell 12: 711–721. 14527416 10.1016/j.molcel.2003.08.010

[42] Suganuma N, Nakamura Y, Yamamoto M, Ohta T, Koiwa H, et al (2003) The Lotus japonicus Sen1 gene controls rhizobial differentiation into nitrogen-fixing bacteroids in nodules. Mol Genet Genomics 269: 312–320. 12684880 10.1007/s00438-003-0840-4

[43] Woolcock KJ, Stunnenberg R, Gaidatzis D, Hotz HR, Emmerth S, et al (2012) RNAi keeps Atf1-bound stress response genes in check at nuclear pores. Genes Dev 26: 683–692. 22431512 10.1101/gad.186866.112 PMC3323879

[44] Linder T, Rasmussen NN, Samuelsen CO, Chatzidaki E, Baraznenok V, et al (2008) Two conserved modules of Schizosaccharomyces pombe Mediator regulate distinct cellular pathways. Nucleic Acids Res 36: 2489–2504. 18310102 10.1093/nar/gkn070 PMC2377428

[45] Zofall M, Yamanaka S, Reyes-Turcu FE, Zhang K, Rubin C, et al (2012) RNA elimination machinery targeting meiotic mRNAs promotes facultative heterochromatin formation. Science 335: 96–100. 22144463 10.1126/science.1211651 PMC6338074

[46] Yamanaka S, Mehta S, Reyes-Turcu FE, Zhuang F, Fuchs RT, et al (2012) RNAi triggered by specialized machinery silences developmental genes and retrotransposons. Nature 493: 557. 23151475 10.1038/nature11716 PMC3554839

[47] Xhemalce B, Kouzarides T (2010) A chromodomain switch mediated by histone H3 Lys 4 acetylation regulates heterochromatin assembly. Genes Dev 24: 647–652. 20299449 10.1101/gad.1881710 PMC2849121

[48] Bahler J, Wu JQ, Longtine MS, Shah NG, McKenzie A 3rd, et al (1998) Heterologous modules for efficient and versatile PCR-based gene targeting in Schizosaccharomyces pombe. Yeast 14: 943–951. 9717240 10.1002/(SICI)1097-0061(199807)14:10<943::AID-YEA292>3.0.CO;2-Y

[49] Motamedi MR, Hong EJ, Li X, Gerber S, Denison C, et al (2008) HP1 proteins form distinct complexes and mediate heterochromatic gene silencing by nonoverlapping mechanisms. Mol Cell 32: 778–790. 19111658 10.1016/j.molcel.2008.10.026 PMC2735125

[50] Tange Y, Kurabayashi A, Goto B, Hoe KL, Kim DU, et al (2012) The CCR4-NOT complex is implicated in the viability of aneuploid yeasts. PLoS Genet 8: e1002776. 22737087 10.1371/journal.pgen.1002776 PMC3380822

[51] Guglielmi B, van Berkum NL, Klapholz B, Bijma T, Boube M, et al (2004) A high resolution protein interaction map of the yeast Mediator complex. Nucleic acids research 32: 5379–5391. 15477388 10.1093/nar/gkh878 PMC524289

