# Supplementary figures and images for: Mediator Directs Co-transcriptional Heterochromatin Assembly by RNA Interference-Dependent and -Independent Pathways
Source: PLoS Genet. 2013 Aug 15;9(8):e1003677. doi: 10.1371/journal.pgen.1003677 (PMC3744440; doi:10.1371/journal.pgen.1003677)

**
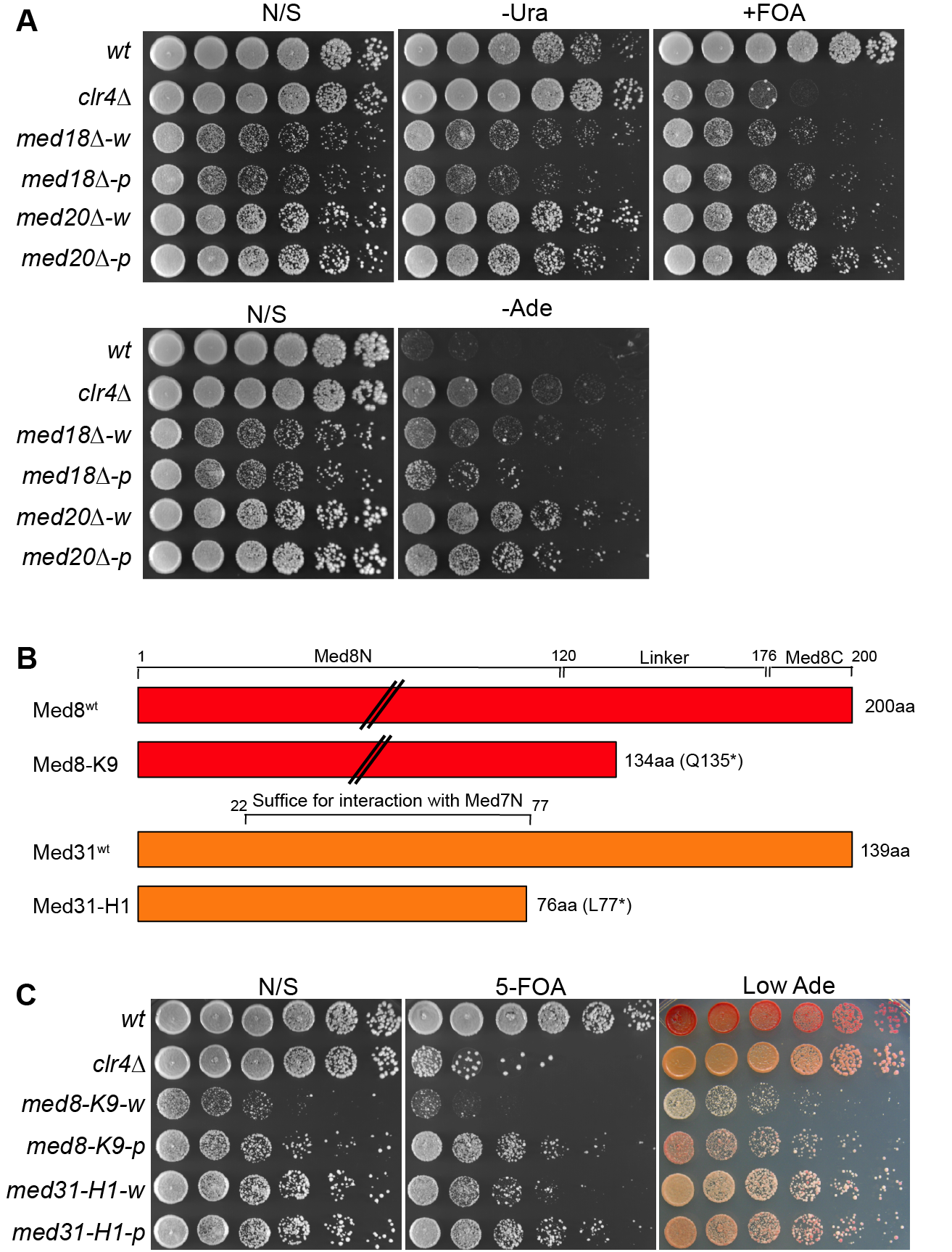
**

Supplement: Figure S1 — Mediator is required for heterochromatin silencing at the pericentromere. (A) Silencing assay at the pericentromere. Shown are the results of serial dilutions of the indicated strains spotted onto non-selective media (N/S), medium without uracil (-Ura), medium with 5-fluoroorotic acid (5-FOA), and medium without adenine (-Ade) to assay ura4+ and ade6+expression. Note that PMGS (EMMS-NH4Cl (nitrogen), +L-glutamic acid, monosodium, as nitrogen) plates were used as N/S plates. (B) Schematic of Med8-K9 and Med31-H1 proteins. med8-K9 contains a point mutation (C540T) causing C-terminal truncation of the Med8 protein (Q135*) (top). The C-terminal residues 176–200 of Med8 (Med8C) are the predicted interface for interaction with Med18 [26], [51]. med31-H1 contains a point mutation (T230A) causing C-terminal truncation of the Med31 protein (L77*) (bottom). Residues 22–77 of Med31 are the predicted interface for interaction with Med7, a core Mediator subunit belonged to the middle domain. (C) Silencing assay at the pericentromere. Shown are the results of serial dilutions of the indicated strains spotted onto non-selective media (N/S), medium with 5-FOA, and medium with a limited amount of adenine (Low Ade) to assay for the presence of imr1L::ura4+ and otr1R::ade6+. (DOC) [file pgen.1003677.s001.doc]

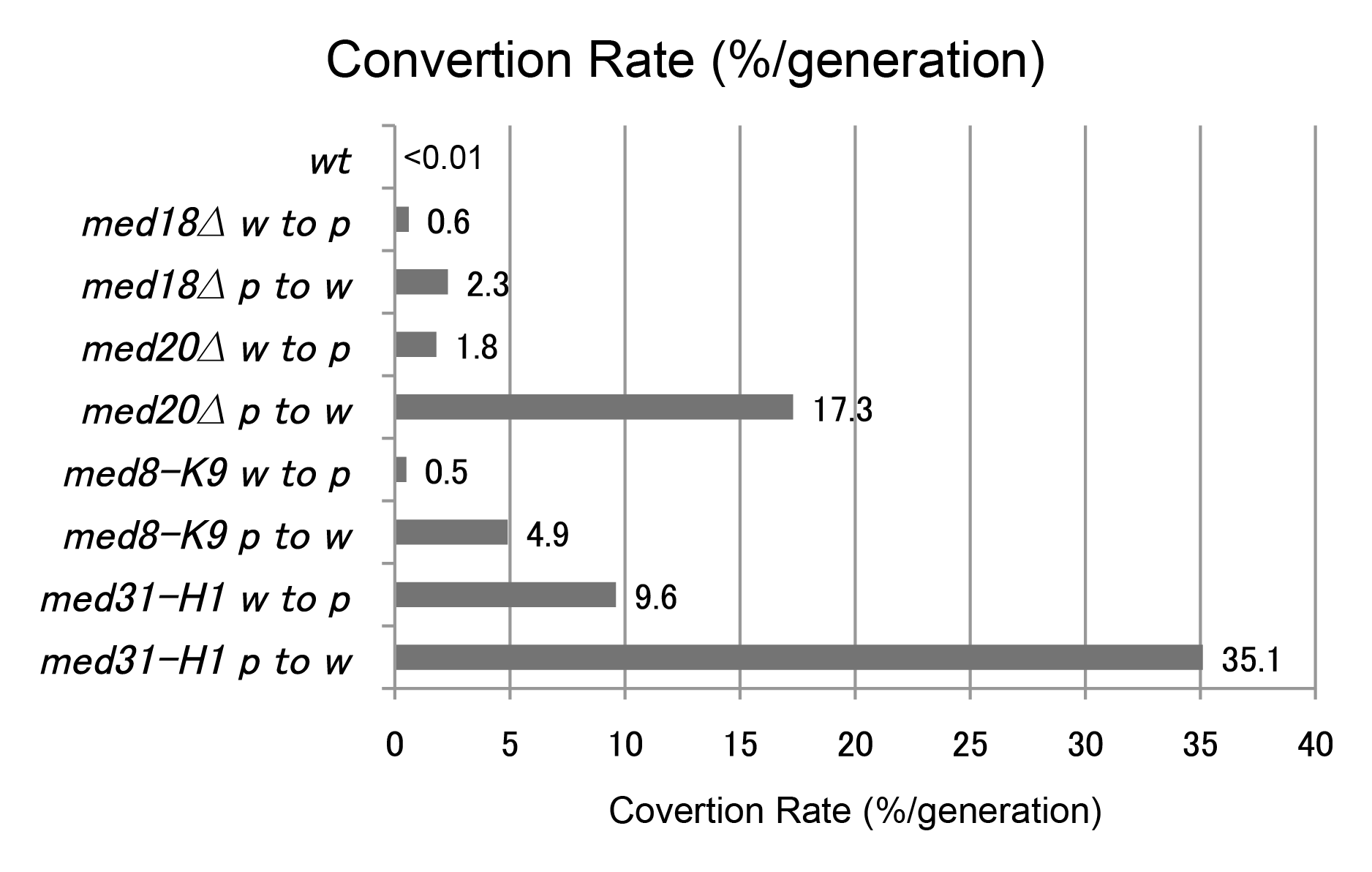

Supplement: Figure S2 — Conversion rates of white/pink epiclones. Stability assay of variegation phenotypes. Conversion rates (percentage of cells that convert the epigenetic state per generation) were measured using indicated strains. w indicates the white epiclones and p indicates the pink epiclones. Cells were grown for several generations. The rates were measured as described in supplemental experimental procedures. (DOC) [file pgen.1003677.s002.doc]

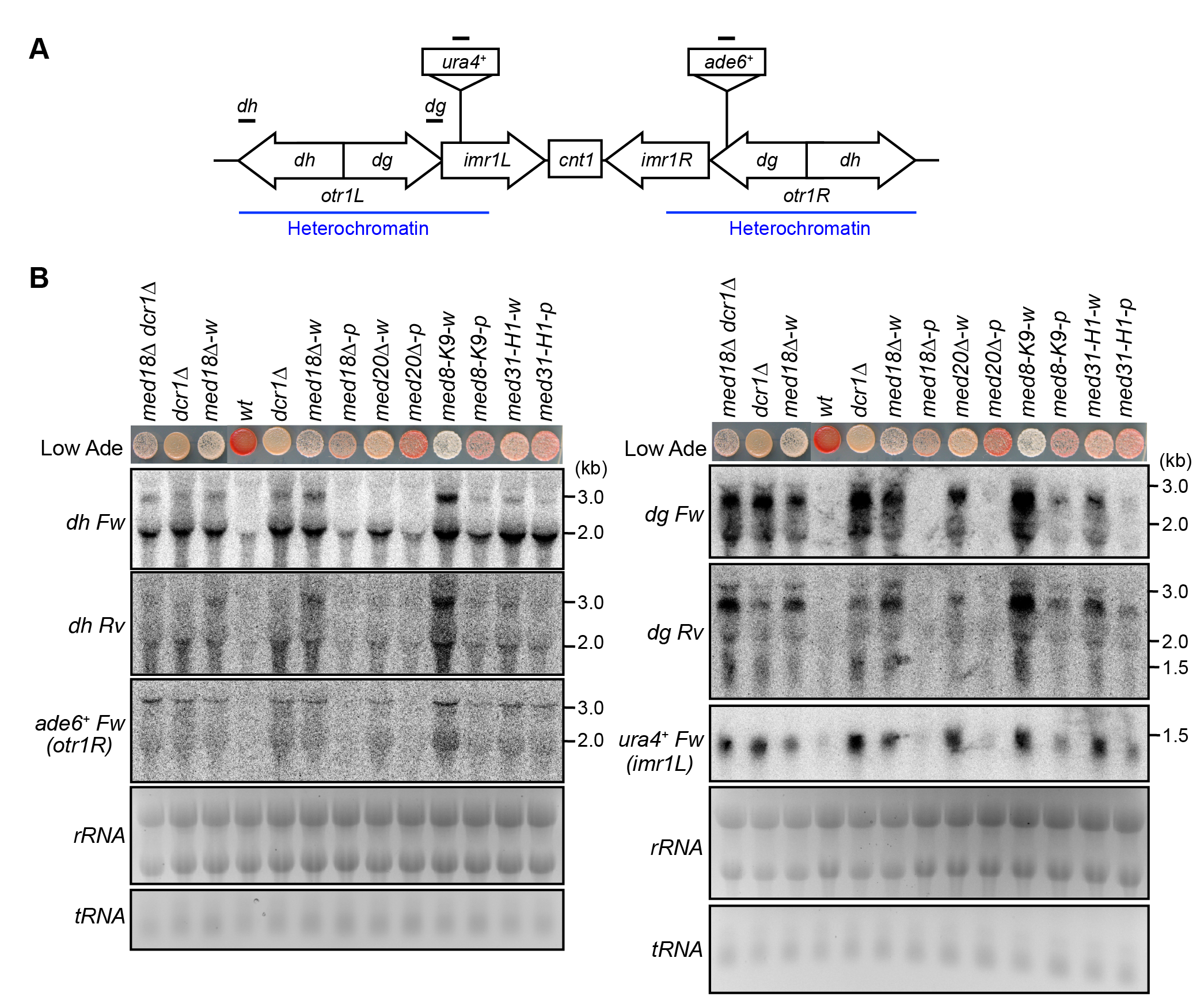

Supplement: Figure S3 — Mediator mutations cause accumulation of centromeric RNA. (A) Schematic of fission yeast centromere 1. Locations of the ura4+ and ade6+ reporters inserted within the pericentromeric region are shown (imr1L::ura4+ and otr1R::ade6+). Black bars indicate the location of probes used for northern analysis. (B) Northern analysis of pericentromeric transcripts in wild-type and mutant cells. Analysis was performed using oligonucleotide probes against dh and dg forward (Fw) and reverse (Rv) strands, imr1L::ura4+ and otr1R::ade6+ forward strand transcripts. rRNA and tRNA were used as loading controls. (DOC) [file pgen.1003677.s003.doc]

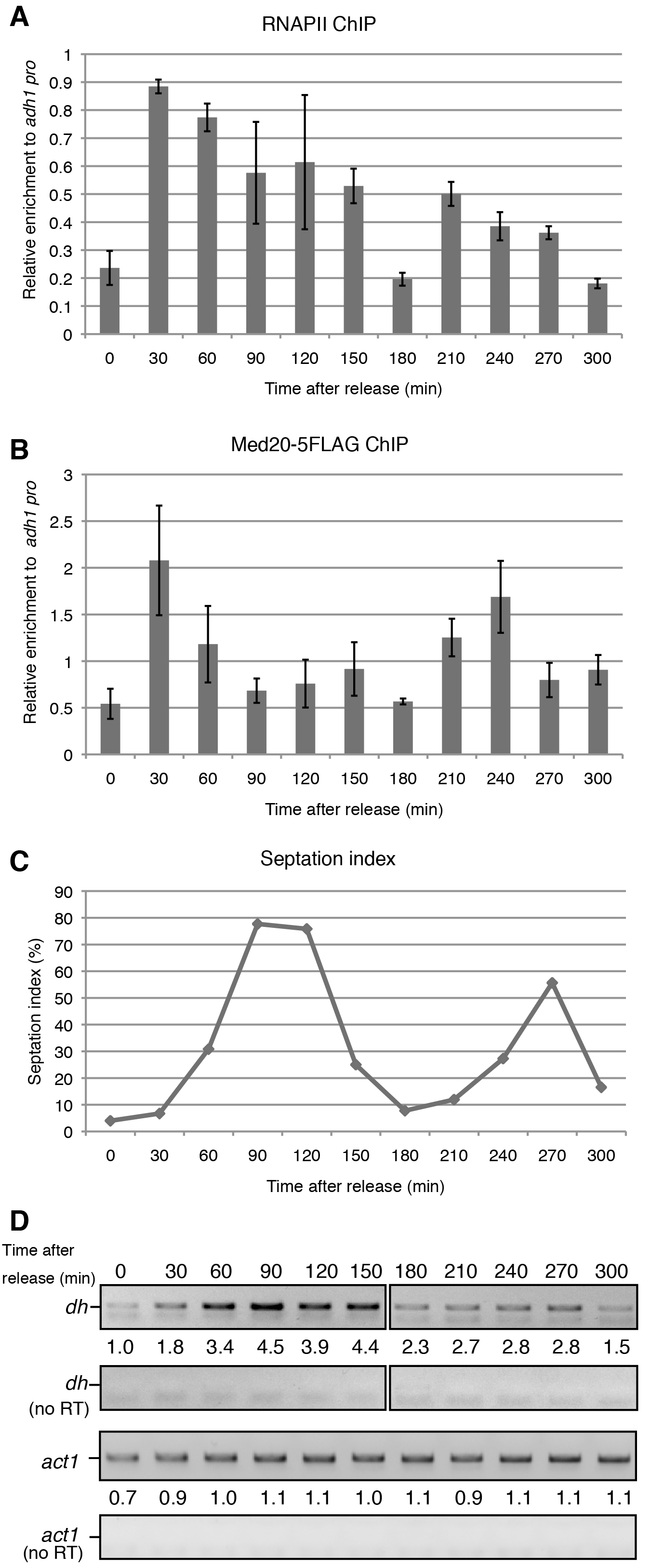

Supplement: Figure S4 — Med20 localizes the pericentromeric heterochromatin with RNAPII in a cell cycle-dependent manner. (A, B) ChIP-qPCR analyses for RNAPII and Med20-5FLAG were performed every 30 minutes after release from G2/M block by cdc25-22 mutation (see Supplemental Experimental Procedures). Enrichment at dh repeats relative to the adh1 promoter region (adh1 pro) is shown. Error bars show the standard error of the mean (n = 3). (C) Septation index (percentage of cells with division septum) was measured to monitor cell cycle progression after release. In a block and release experiment, the peak of septation (90 to 120 min) indicates S phase. (D) RT-PCR of dh transcripts was performed as described in A and B. Transcripts derived from constitutively expressed act1 served as controls. “No RT” indicates that no reverse transcriptase was added in the reaction. Numbers under the panels of dh and act1 indicate the increase in transcript relative to the values at 0 min (dh) and 60 min (act1), respectively. (TIF) [file pgen.1003677.s004.tif]

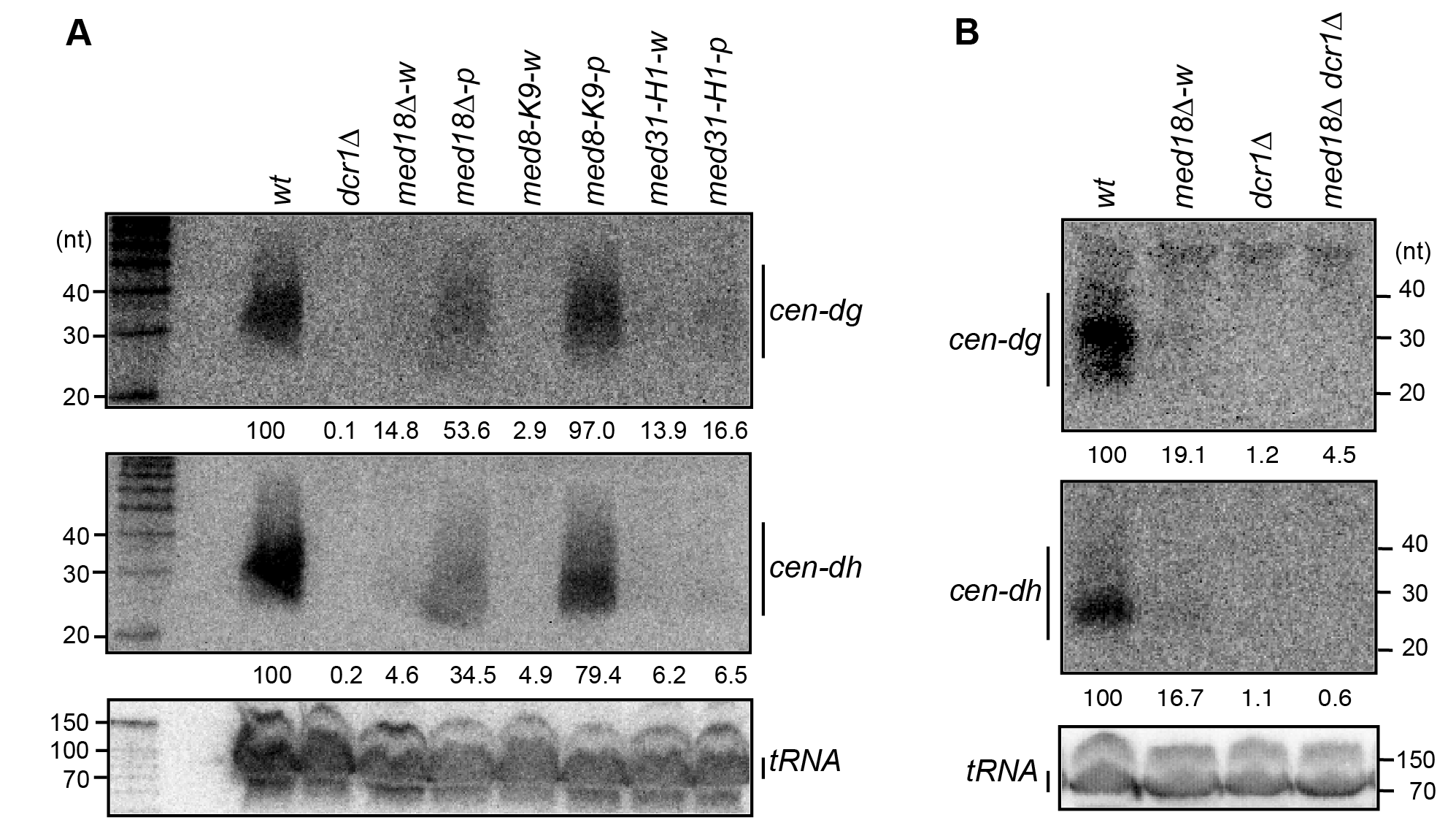

Supplement: Figure S5 — Northern analysis of siRNA in the Mediator mutants med8-K9 and med31-H1. Analyses were performed with RNA isolated from the indicated strains (panel A: single deletion mutants, panel B: single and double deletion mutants of dcr1 and med18) using oligonucleotide probes specific for dg/dh centromeric repeats. tRNA was used as a loading control. (DOC) [file pgen.1003677.s005.doc]

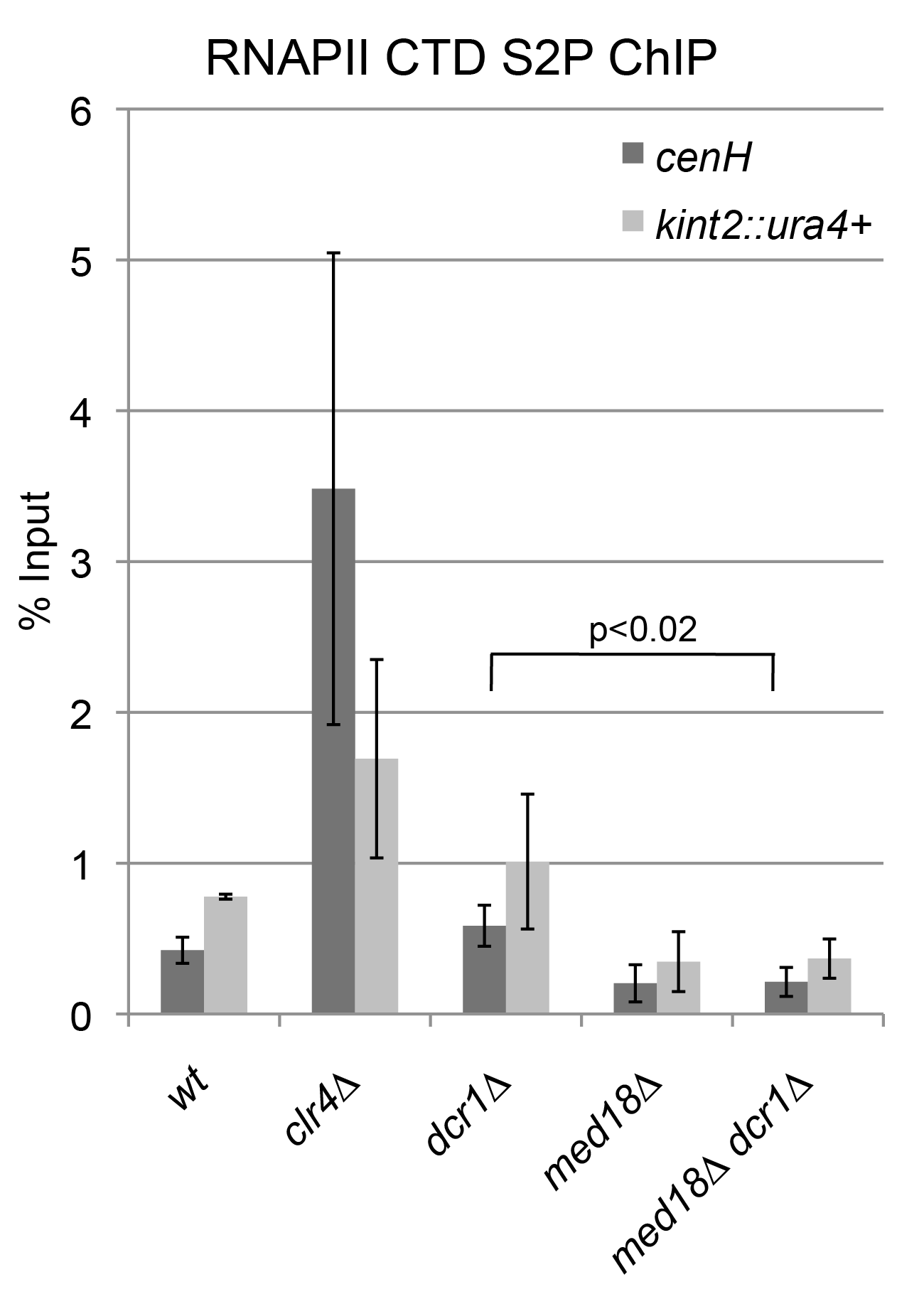

Supplement: Figure S6 — Mediator is required for transcriptional activation in heterochromatin. ChIP analysis of RNAPII at cenH dh repeats or kint2::ura4+ relative to input WCE in the indicated strains. Anti-RNA polymerase II C-terminal domain (CTD) serine 2 phosphorylation was used. Error bars represent the standard error of the mean (n = 3). P values were determined using a two-sided Student's t-test. (TIF) [file pgen.1003677.s006.tif]

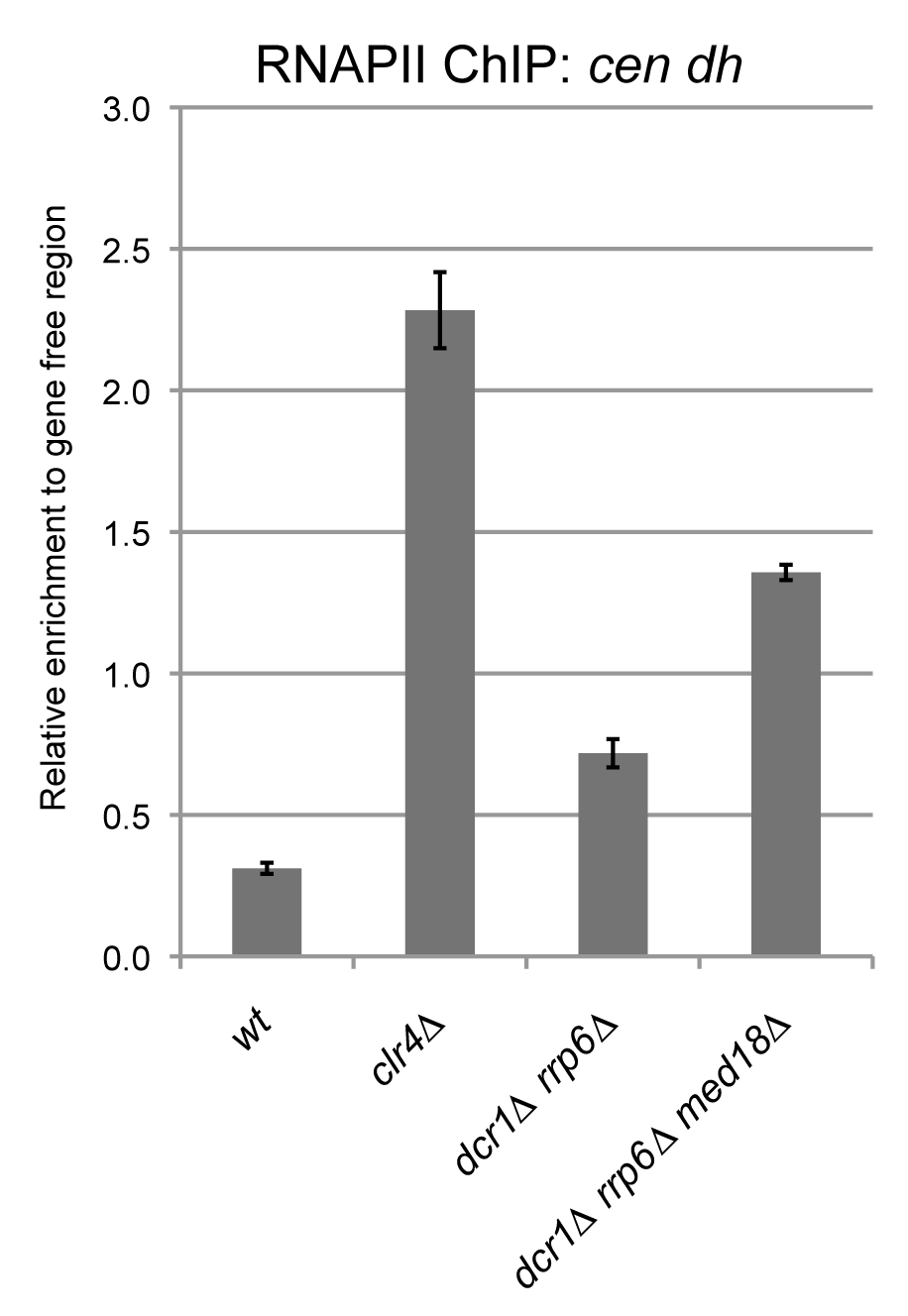

Supplement: Figure S7 — Mediator negatively regulates RNAPII in the compromised heterochromatin. ChIP analysis of RNAPII at dh repeats relative to the gene free region in the indicated strains. Error bars show the standard error of the mean (n = 3). (TIF) [file pgen.1003677.s007.tif]

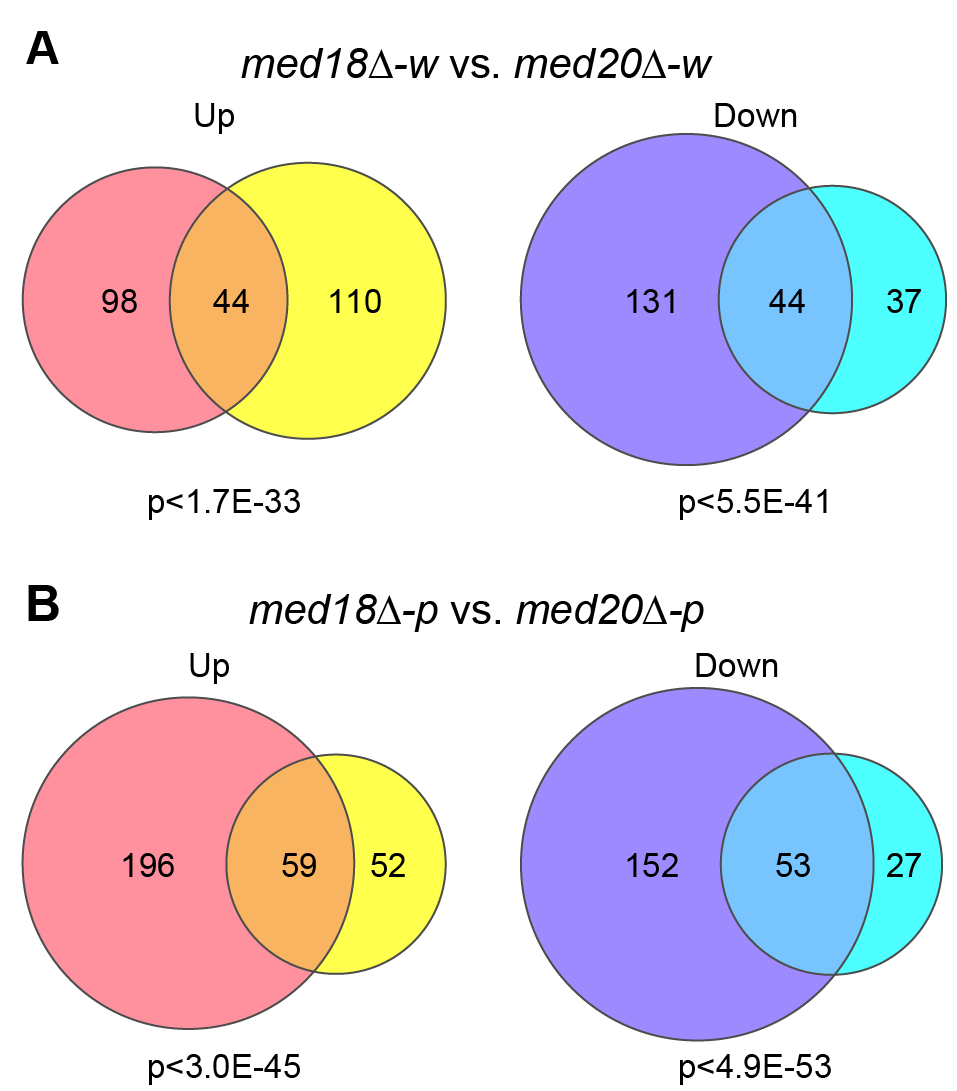

Supplement: Figure S8 — Effect of Mediator disruption on euchromatic genes. Venn diagram showing the number of transcripts whose expression levels were increased (up) or decreased (down) >1.5-fold in mutants compared to the wild type. The p-value was calculated using Fisher's exact test. (A) Transcripts of med18Δ-w (left circles) vs. med20Δ-w (right circles) mutants. (B) Transcripts of med18Δ-p (left circles) vs. med20Δ-p (right circles) mutants. (DOC) [file pgen.1003677.s008.doc]
