# Supplementary material for: Mediator Directs Co-transcriptional Heterochromatin Assembly by RNA Interference-Dependent and -Independent Pathways
Source: PLoS Genet. 2013 Aug 15;9(8):e1003677. doi: 10.1371/journal.pgen.1003677 (PMC3744440; doi:10.1371/journal.pgen.1003677)
Supplement: Table S1 — Strains used in this study. (DOCX) [file pgen.1003677.s009.docx]

**Table S1. Strains used in this study**

| Name | Genotype | Source |
| --- | --- | --- |
| FY2002 | *h^+^, leu1-32, ade6-DN/N, ura4-DS/E, imr1L::ura4^+^, otr1R::ade6^+^* | R. Allshire |
| HKV-324 | *h^+^, leu1-32, ade6-DN/N, ura4-DS/E, imr1L::ura4^+^, otr1R::ade6^+^, clr4∆::kanMX6* | our stock |
| HKV-325 | *h^+^, leu1-32, ade6-DN/N, ura4-DS/E, imr1L::ura4^+^, otr1R::ade6^+^, dcr1∆::kanMX6* | our stock |
| HKV-435 | *h^+^, leu1-32, ade6-DN/N, ura4-DS/E, imr1L::ura4^+^, otr1R::ade6^+^, med1∆::kanMX6* | this study |
| HKV-389 | *h^+^, leu1-32, ade6-DN/N, ura4-DS/E, imr1L::ura4^+^, otr1R::ade6^+^, med27∆::kanMX6* | this study |
| HKV-391 | *h^+^, leu1-32, ade6-DN/N, ura4-DS/E, imr1L::ura4^+^, otr1R::ade6^+^, med18∆::kanMX6* | this study |
| EOS-164 | *h^+^, leu1-32, ade6-DN/N, ura4-DS/E, imr1L::ura4^+^, otr1R::ade6^+^, med20∆::kanMX6* | this study |
| HKV-393 | *h^+^, leu1-32, ade6-DN/N, ura4-DS/E, imr1L::ura4^+^, otr1R::ade6^+^, med19∆::kanMX6* | this study |
| HKV-395 | *h^+^, leu1-32, ade6-DN/N, ura4-DS/E, imr1L::ura4^+^, otr1R::ade6^+^, med12∆::kanMX6* | this study |
| HKV-397 | *h^+^, leu1-32, ade6-DN/N, ura4-DS/E, imr1L::ura4^+^, otr1R::ade6^+^, med13∆::kanMX6* | this study |
| HKV-399 | *h^+^, leu1-32, ade6-DN/N, ura4-DS/E, imr1L::ura4^+^, otr1R::ade6^+^, cdk8∆::kanMX6* | this study |
| EOS-071 | *h^+^, leu1-32, ade6-DN/N, ura4-DS/E, imr1L::ura4^+^, otr1R::ade6^+^, med18∆::kanMX6 (white epiclone)* | this study |
| EOS-073 | *h^+^, leu1-32, ade6-DN/N, ura4-DS/E, imr1L::ura4^+^, otr1R::ade6^+^, med18∆::kanMX6 (pink epiclone)* | this study |
| EOS-205 | *h^+^, leu1-32, ade6-DN/N, ura4-DS/E, imr1L::ura4^+^, otr1R::ade6^+^, med20∆::kanMX6 (white epiclone)* | this study |
| EOS-206 | *h^+^, leu1-32, ade6-DN/N, ura4-DS/E, imr1L::ura4^+^, otr1R::ade6^+^, med20∆::kanMX6 (pink epiclone)* | this study |
| SPY797 | *h^+^, leu1-32, ade6-m210, ura4-DS/E, otr1R(SphI)::ura4^+^, Nat-Ago1promoter-3×FLAG::ago1* | D. Moazed |
| KKS-690 | *h^+^, leu1-32, ade6-m210, ura4-DS/E, otr1R(SphI)::ura4^+^, Nat-Ago1promoter-3×FLAG::ago1, clr4∆::hphMX6* | our stock |
| KKS-688 | *h^+^, leu1-32, ade6-m210, ura4-DS/E, otr1R(SphI)::ura4^+^, Nat-Ago1promoter-3×FLAG::ago1, dcr1∆::hphMX6* | our stock |
| EOS-624 | *h^+^, leu1-32, ade6-m210, ura4-DS/E, otr1R(SphI)::ura4^+^, Nat-Ago1promoter-3×FLAG::ago1, med18∆::kanMX6* | this study |
| FY648 | *h^+^, leu1-32 , ade6-m210, ura4-DS/E, otr1R(SphI)::ura4^+^* | R. Allshire |
| KKS-341 | *h^+^, leu1-32, ade6-DN/N, ura4-DS/E, imr1L::ura4^+^, otr1R::ade6^+^, chp1-13myc-kanMX6* | our stock |
| EOS-654 | *h^+^, leu1-32, ade6-DN/N, ura4-DS/E, imr1L::ura4^+^, otr1R::ade6^+^, chp1-13myc-kanMX6, clr4∆::hphMX6* | this study |
| EOS-656 | *h^+^, leu1-32, ade6-DN/N, ura4-DS/E, imr1L::ura4^+^, otr1R::ade6^+^, chp1-13myc-kanMX6, dcr1∆::hphMX6* | this study |
| EOS-650 | *h^+^, leu1-32, ade6-DN/N, ura4-DS/E, imr1L::ura4^+^, otr1R::ade6^+^, chp1-13myc-kanMX6, med18∆::natMX6 (white epiclone)* | this study |
| EOS-652 | *h^+^, leu1-32, ade6-DN/N, ura4-DS/E, imr1L::ura4^+^, otr1R::ade6^+^, chp1-13myc-kanMX6, med18∆::natMX6 (pink epiclone)* | this study |
| KKS-357 | *h^+^, leu1-32, ade6-DN/N, ura4-DS/E, imr1L::ura4^+^, otr1R::ade6^+^, rdp1-5FLAG-natMX6* | our stock |
| EOS-660 | *h^+^, leu1-32, ade6-DN/N, ura4-DS/E, imr1L::ura4^+^, otr1R::ade6^+^, rdp1-5FLAG-natMX6, clr4∆::hphMX6* | this study |
| EOS-662 | *h^+^, leu1-32, ade6-DN/N, ura4-DS/E, imr1L::ura4^+^, otr1R::ade6^+^, rdp1-5FLAG-natMX6, dcr1∆::hphMX6* | this study |
| EOS-700 | *h^+^, leu1-32, ade6-DN/N, ura4-DS/E, imr1L::ura4^+^, otr1R::ade6^+^, rdp1-5FLAG-natMX6, med18∆::hphMX6 (white epiclone)* | this study |
| EOS-702 | *h^+^, leu1-32, ade6-DN/N, ura4-DS/E, imr1L::ura4^+^, otr1R::ade6^+^, rdp1-5FLAG-natMX6, med18∆::hphMX6 (pink epiclone)* | this study |
| SPY440 | *h^-^, ura4-5BoxB-hphMX6* | D. Moazed |
| SPY452 | *h^-^, ura4-5BoxB-hphMX6, tas3-λN-kanMX6* | D. Moazed |
| EOS-469 | *h^-^, ura4-5BoxB-hphMX6, tas3-λN-kanMX6, FOA^r^* | this study |
| SPY463 | *h^-^, ura4-5BoxB-hphMX6, tas3-λN-kanMX6, clr4∆::natMX6* | D. Moazed |
| EOS-544 | *h^-^, ura4-5BoxB-hphMX6, tas3-λN-kanMX6, med18∆::natMX6* | this study |
| EOS-541 | *h^-^, ura4-5BoxB-hphMX6, tas3-λN-kanMX6, med20∆::natMX6* | this study |
| HKV-174 | *h^90^, his2, leu1-32, ura4-DS/E, ade6-m210, kint2::ura4^+^* | our stock |
| HKV-171 | *h^90^, his2, leu1-32, ura4-DS/E, ade6-m210, kint2::ura4^+^, clr4Δ::kanMX6* | our stock |
| HKV-320 | *h^90^, his2, leu1-32, ura4-DS/E, ade6-m210, kint2::ura4^+^, dcr1Δ::kanMX6* | our stock |
| EOS-391 | *h^90^, his2, leu1-32, ura4-DS/E, ade6-m210, kint2::ura4^+^, med18Δ::kanMX6* | this study |
| EOS-643 | *h90, his2, leu1-32, ura4-DS/E, ade6-m210, kint2::ura4^+^, med18Δ::kanMX6, dcr1∆::hphMX6* | this study |
| EOS-012 | *h^-^* | this study |
| GAS-28 | *h^-^, clr4∆::kanMX6* | our stock |
| EOS-426 | *h^-^, ade6-DN/N, ura4-DS/E, imr1L::ura4^+^, otr1R::ade6^+^, med18∆::hphMX6, dcr1∆::kanMX6* | this study |
| EOS-695 | *h^90^, his2, leu1-32, ura4-DS/E, ade6-m210, kint2::ura4^+^, rrp6Δ::natMX6* | this study |
| EOS-694 | *h^90^, his2, leu1-32, ura4-DS/E, ade6-m210, kint2::ura4^+^, dcr1Δ::kanMX6, rrp6Δ::natMX6* | this study |
| EOS-704 | *h^90^, his2, leu1-32, ura4-DS/E, ade6-m210, kint2::ura4^+^, med18Δ::kanMX6, rrp6∆::natMX6* | this study |
| EOS-706 | *h^90^, his2, leu1-32, ura4-DS/E, ade6-m210, kint2::ura4^+^, med18Δ::kanMX6, dcr1∆::hphMX6, rrp6Δ::natMX6* | this study |
| HKM-1374 | *h^+^, leu1-32, ade6-DN/N, ura4-DS/E, imr1L::ura4^+^, otr1R::ade6^+^, clr4∆::kanMX6, med8-K9* | this study |
| EOS-599 | *h^+^, leu1-32, ade6-DN/N, ura4-DS/E, imr1L::ura4^+^, otr1R::ade6^+^, med8-K9 (white epiclone)* | this study |
| EOS-601 | *h^+^, leu1-32, ade6-DN/N, ura4-DS/E, imr1L::ura4^+^, otr1R::ade6^+^, med8-K9 (pink epiclone)* | this study |
| HKM-1369 | *h^+^, leu1-32, ade6-DN/N, ura4-DS/E, imr1L::ura4^+^, otr1R::ade6^+^, med31-H1* | this study |
| EOS-586 | *h^+^, leu1-32, ade6-DN/N, ura4-DS/E, imr1L::ura4^+^, otr1R::ade6^+^, med31-H1(white epiclone)* | this study |
| EOS-588 | *h^+^, leu1-32, ade6-DN/N, ura4-DS/E, imr1L::ura4^+^, otr1R::ade6^+^, med31-H1(pink epiclone)* | this study |
| ss216 | *h^+^, leu1-32, ade6-DN/N, ura4-DS/E, imr1L::ura4^+^, otr1R::ade6^+^, rrp6-13myc-natMX6, dcr1∆::hphMX6* | Our stock |
| EOS-747 | *h^+^, leu1-32, ade6-DN/N, ura4-DS/E, imr1L::ura4^+^, otr1R::ade6^+^, rrp6-13myc-natMX6, clr4∆::kanMX6* | this study |
| EOS-739 | *h^+^, leu1-32, ade6-DN/N, ura4-DS/E, imr1L::ura4^+^, otr1R::ade6^+^, rrp6-13myc-natMX6, dcr1∆::hphMX6* | this study |
| EOS-788 | *h^+^, leu1-32, ade6-DN/N, ura4-DS/E, imr1L::ura4^+^, otr1R::ade6^+^, rrp6-13myc-natMX6, med18∆::hphMX6 (white epiclone)* | this study |
| EOS-789 | *h^+^, leu1-32, ade6-DN/N, ura4-DS/E, imr1L::ura4^+^, otr1R::ade6^+^, rrp6-13myc-natMX6, med18∆::hphMX6 (pink epiclone)* | this study |
| EOS-797 | *h^+^, leu1-32, ade6-DN/N, ura4-DS/E, imr1L::ura4^+^, otr1R::ade6^+^, rrp6-13myc-natMX6, dcr1∆::hphMX6, med18∆::hphMX6* | this study |
| EOS-588 | *h^+^, leu1-32, ade6-DN/N, ura4-DS/E, imr1L::ura4^+^, otr1R::ade6^+^, med31-H1(pink epiclone)* | this study |
