# Supplementary material for: Mediator Directs Co-transcriptional Heterochromatin Assembly by RNA Interference-Dependent and -Independent Pathways
Source: PLoS Genet. 2013 Aug 15;9(8):e1003677. doi: 10.1371/journal.pgen.1003677 (PMC3744440; doi:10.1371/journal.pgen.1003677)
Supplement: Table S2 — Primers used in this study. (DOCX) [file pgen.1003677.s010.docx]

**Table S2. Primers used in this study**

| Primer ID | Name | Sequence | Used for |
| --- | --- | --- | --- |
| EOO-042 | pFA6a Fw | TCGTACGCTGCAGGTCGACGGATCCCC | amplification of marker cassette |
| EOO-043 | pFA6a Rv | ATCGATGAATTCGAGCTCGTTTAAAC | amplification of marker cassette |
| EOO-139 | dh Fw | CTCTCATCTCGACTCGTTTG | ChIP, RT-PCR |
| EOO-140 | dh Rv | GGCATTCACGAAACATAGCG | ChIP, RT-PCR |
| EOO-482 | ade6 Fw | GTAGTACGCAGTTTAGACGG | ChIP |
| EOO-483 | ade6 Rv | GAGCACGCTGTTGAATTGAG | ChIP, Northern analysis |
| EOO-484 | ura4 Fw | GAATGGTTTGAGAAGCATACC | ChIP |
| EOO-485 | ura4 Rv | GAGTACGATATTGCTGTCCC | ChIP, Northern analysis |
| EOS-141 | act1 Fw | TGCCGATCGTATGCAAAAGG | ChIP, RT-PCR |
| EOS-142 | act1 Rv | CCGCTCTCATCATACTCTTG | ChIP, RT-PCR |
| KKO-480 | fbp1 Fw | GTCGAACGGATGCTGCAAAC | ChIP |
| KKO-481 | fbp1 Rv | GGTACCTACACTAACACCGG | ChIP |
| KKO-482 | adh1 Fw | GGTGTCAAGTGGATGAACTC | ChIP |
| KKO-483 | adh1 Rv | GCATTGGCAATGCAGTAGTG | ChIP |
| EOS-451 | SPCTRNAASN.05 Fw | AATATATCAAGCAAGAATGGGG | ChIP |
| EOS-450 | SPCTRNAASN.05 Rv | CGACCTCACGATTAACAGTCG | ChIP, siRNA analysis |
| EOS-513 | gene free region Fw | CAGTGGTAAGGGATTGTTGTAAGG | ChIP |
| EOS-514 | gene free region Rv | TGGCATAGAGGACGGAAGG | ChIP |
| EOS-529 | cenH dh Fw | GCTAAGATCGATTGGTGACG | ChIP, RT-PCR |
| EOS-530 | cenH dh Rv | AAGTTCACTGTTCTTATACACTGG | ChIP, RT-PCR |
| EOS-495 | SPRRNA.48 Fw | AACAGCCTCTAGTGCAGATC | ChIP, RT-PCR |
| EOS-496 | SPRRNA.48 Rv | GAGCTTCCCTATCTCTTAGG | ChIP, RT-PCR |
| EOO-517 | dh Fw | ACAACGCATCTACCTCAGCAGTCCTTGGG | Northern analysis |
| EOO-518 | dh Rv | CCCAAGGACTGCTGAGGTAGATGCGTTGT | Northern analysis |
| EOO-519 | dg Rv | CCATCCGCAGTTGGGAGTACATCATTCC | Northern analysis |
| EOO-520 | dg Fw | GGAATGATGTACTCCCAACTGCGGATGG | Northern analysis |
| KKO-586 | cen siRNA A | GCGACTAAACCGAAAGCCTC | siRNA analysis |
| KKO-587 | cen siRNA B | TACCGTGATTAGCCTTACTCCGCATT | siRNA analysis |
| KKO-588 | cen siRNA C | TACTTATTGATGGCGAAGCTAGA | siRNA analysis |
| KKO-589 | cen siRNA D | TACCGCTTCTCCTTAATCCA | siRNA analysis |
| KKO-590 | cen siRNA E | ACACCTACTCTTATCACTTGT | siRNA analysis |
| KKO-591 | cen siRNA F | GACGATAAGCAGGAGTTGCGCA | siRNA analysis |
| KKO-592 | cen siRNA G | AGTGTGGCGCTATATCTTGTA | siRNA analysis |
| KKO-593 | cen siRNA H | TACTGTCATTAGGATATGCTCA | siRNA analysis |
| KKO-594 | cen siRNA I | GGGAAATGTATAAATAGGCA | siRNA analysis |
| KKO-595 | cen siRNA J | TTTCCCAAGGACTGCTGAGGTAGA | siRNA analysis |
| KKO-596 | cen siRNA K | TGGACACAGCATGGATATGGACACA | siRNA analysis |
| KKO-597 | cen siRNA L | TGGCAGATATTGCAAGTTGTTTA | siRNA analysis |
| KKO-623 | cen siRNA-1 | CCTTAATTAAAAACGACCAATATG | siRNA analysis |
| KKO-624 | cen siRNA-2 | CTGCGGTTCACCCTTAACATC | siRNA analysis |
| KKO-625 | cen siRNA-3 | CAACTGCGGATGGAAAAAGT | siRNA analysis |
| KKO-626 | cen siRNA-4 | CTCTATATATCAGATATAAAGATGCG | siRNA analysis |
| KKO-627 | cen siRNA-5 | GTTTTGAAGTAGACATTCCGCACAA | siRNA analysis |
| KKO-628 | cen siRNA-6 | CTGAGCACAAGAGACATGGTGTACTAGA | siRNA analysis |
| KKO-629 | cen siRNA-7 | GTACATTTTTGCAGGACAACCAG | siRNA analysis |
| KKO-630 | cen siRNA-8 | CAACAACAGTCTTGGATTTATTTAG | siRNA analysis |
| EOO-529 | cenH dh Rv | GCTAAGATCGATTGGTGACG | ChIP |
| EOO-530 | cenH dh Fw | AAGTTCACTGTTCTTATACACTGG | ChIP |
| EOO-219 | adh1 promoter Fw | TCTCATTGGTCTTCCGCTCC | ChIP-qPCR time course analysis |
| EOO-220 | adh1 promoter Rv | AAGAAAAGCGAAGGCACCTG | ChIP-qPCR time course analysis |
