# Supplementary material for: Mediator Directs Co-transcriptional Heterochromatin Assembly by RNA Interference-Dependent and -Independent Pathways
Source: PLoS Genet. 2013 Aug 15;9(8):e1003677. doi: 10.1371/journal.pgen.1003677 (PMC3744440; doi:10.1371/journal.pgen.1003677)
Supplement: Text S1 — Supplemental methods. (DOCX) [file pgen.1003677.s011.docx]

**Text S1**

**Supplemental Methods**

ChIP-qPCR and RT-PCR using synchronized *cdc25-22* cells.

*cdc25-22* cells were grown at 25ºC to a concentration of 2×10^6^ cells/ml and then shifted to 36ºC for 4 hr and 15 min to stop the cell cycle at the G2/M phase. Samples for ChIP assay were collected every 30 min for 300 min after shifting the cells back to 25ºC to release cell cycle block. ChIP assay was performed as described in the Experimental Procedures. To prepare RNA for RT-PCR, the input fractions of ChIP were adjusted to 0.25% SDS and 0.25 mg/ml proteinase K and incubated for 45 min at 45ºC and then at 65ºC for 4 hours to reverse crosslinking. Samples were extracted once with phenol-chloroform. After ethanol precipitation, the samples were resuspended in a suitable volume of DEPC-treated distilled water. RT-PCR was performed as described in the Experimental Procedures.

Stability assay of variegation phenotypes

White or pink epiclones on Low Ade plates (YES plates including limited amount of adenine) were cultured for several generations in complete medium. Aliquots were taken from the cultures before and after the cultivation, appropriately diluted, and then plated onto Low Ade plates and incubated at 30ºC for several days to allow white or pink colonies to form. Conversion rates were calculated using the following formula: conversion rate = 1- (*F/I*) ^1/^*^N^*, where *F* is the final percentage of white or pink colonies, *I* is the initial percentage of white or pink colonies, and *N* is the number of generations between *I* and *F* [1,2].

**References for supplemental methods**

1. Kipling D, Kearsey SE (1990) Reversion of autonomously replicating sequence mutations in Saccharomyces cerevisiae: creation of a eukaryotic replication origin within prokaryotic vector DNA. Molecular and cellular biology **10:** 265-272

2. Nakagawa H, Lee JK, Hurwitz J, Allshire RC, Nakayama J, Grewal SI, Tanaka K, Murakami Y (2002) Fission yeast CENP-B homologs nucleate centromeric heterochromatin by promoting heterochromatin-specific histone tail modifications. Genes & development **16:** 1766-1778
